# Evidence Mapping, Certainty Appraisal, and Acupoint Prescription Pattern Mining of Acupuncture for Poststroke Dysphagia: An Overview of Systematic Reviews

**DOI:** 10.1002/brb3.71887

**Published:** 2026-11-01

**Authors:** Guohao Lan, Minmin Wu, Jiongliang Zhang, Yuxuan Li, Yuting Wang, Yumeng Su, Zhiliang Dong, Xiangyu Wei, Luwen Zhu

**Affiliations:** ^1^ Heilongjiang University of Chinese Medicine Harbin Heilongjiang China; ^2^ Department of Acupuncture The Second Affiliated Hospital of Heilongjiang University of Chinese Medicine Harbin Heilongjiang China; ^3^ Rehabilitation Center The Second Affiliated Hospital of Heilongjiang University of Chinese Medicine Harbin Heilongjiang China; ^4^ Heilongjiang Provincial Key Laboratory of Brain Function and Neurorehabilitation The Second Affiliated Hospital of Heilongjiang University of Chinese Medicine Harbin Heilongjiang China

**Keywords:** acupuncture, evidence synthesis, neurological rehabilitation, poststroke dysphagia

## Abstract

**Background:**

To characterize the methodological quality, certainty of evidence, primary‐study overlap, outcome coverage, and recurrent acupuncture prescription patterns in the existing secondary evidence on acupuncture for poststroke dysphagia (PSD).

**Method:**

Systematic reviews and meta‐analyses (SRs/MAs) evaluating acupuncture for PSD were identified. Methodological quality was assessed using A Measurement Tool to Assess Systematic Reviews 2 (AMSTAR 2), certainty of evidence using the Grading of Recommendations Assessment, Development and Evaluation (GRADE) approach, and primary‐study overlap using the corrected covered area (CCA). Evidence mapping summarized outcome coverage and certainty, and prescription pattern mining explored frequently used acupoints and combinations.

**Result:**

Forty‐five SRs/MAs were included. Under the original AMSTAR 2 critical‐domain configuration, one review was rated moderate, eight low, and 36 critically low. GRADE indicated that most outcome evidence was of low or very low certainty. The CCA was 0.34%, indicating slight overlap of primary studies across reviews. Evidence was concentrated on total effective rate and swallowing‐function outcomes, whereas patient‐important outcomes, including aspiration/pneumonia, nutritional status, safety, and quality of life, were underrepresented. Frequently used acupoints included Fengchi (GB20), Lianquan (RN23), Jinjin (EX‐HN12), and Yuye (EX‐HN13), with recurrent combinations reflecting local stimulation and swallowing‐related functional regulation.

**Conclusion:**

Current secondary evidence suggests that acupuncture, primarily as an adjunctive intervention, may improve swallowing‐related outcomes in PSD, but confidence remains limited by methodological weaknesses and low certainty of evidence. Future studies should improve methodological rigor, standardize clinically important outcomes, and prospectively validate recurrent prescription patterns.

## Graphical abstract

This overview mapped evidence from 45 systematic reviews/meta‐analyses of acupuncture for post‐stroke dysphagia and appraised quality, certainty, overlap, and prescription patterns. Findings suggest potential adjunctive benefits but limited confidence, highlighting the need for rigorous trials and standardized swallowing‐related outcomes.

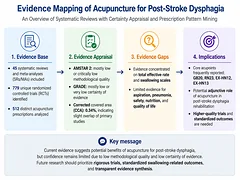

## 1 Introduction

Stroke is one of the leading causes of death and disability worldwide, with epidemiological data showing that 12.2 million incident stroke cases occurred globally in 2019. Stroke can cause multisystem functional impairment and substantially reduce patients' quality of life (Feigin et al. 2021). Among its complications, poststroke dysphagia (PSD) is a common and clinically consequential condition that may occur in the acute phase and persist into the rehabilitation phase. Epidemiological estimates indicate that the incidence of PSD varies according to the assessment method used, ranging from 37% to 78% (Martino et al. 2005). PSD not only contributes to malnutrition but also markedly increases the risk of aspiration, which has been reported in approximately 43%–54% of affected patients. Aspiration, in turn, has been associated with a threefold increase in the risk of aspiration pneumonia and a substantial increase in mortality (Li et al. 2024).

Currently, therapeutic strategies for PSD mainly include pharmacotherapy, swallowing rehabilitation, physical therapy, and biofeedback. However, responses to conventional rehabilitation remain limited in some patients, and treatment often requires a prolonged course; therefore, adjunctive interventions that may improve swallowing recovery and rehabilitation outcomes are still clinically needed (Zhu et al. 2024; Feng et al. 2016). Acupuncture has been proposed as a potential adjunctive approach for enhancing the swallowing reflex, promoting muscular coordination, and facilitating neurological recovery (Liu et al. 2021; Zhang et al. 2025). Some clinical studies have suggested that acupuncture combined with conventional rehabilitation may improve swallowing function and reduce the risk of complications (Zhang et al. 2024; Qiu et al. 2022). Previous systematic reviews have also indicated that acupuncture may improve swallowing function and related quality of life, although the certainty of the evidence remains uncertain (Wang et al. 2022). Despite the increasing number of systematic reviews and meta‐analyses (SRs/MAs) in this area, the existing evidence shows inconsistencies in study design, reporting quality, comparator selection, and outcome selection and measurement. These inconsistencies limit the ability of this evidence to inform neurological rehabilitation practice. Against this background, a systematic overview of the overall structure and distribution of evidence in this field is warranted.

The Global Evidence Mapping (GEM) initiative was originally established to provide systematic overviews for broad research areas such as traumatic brain injury and spinal cord injury (Bragge et al. 2011). Evidence mapping, which emerged from this methodological context, has become an established approach for organizing and visualizing the distribution of available studies. It is designed to characterize the research landscape, identify knowledge gaps, and provide an overarching appraisal of a field alongside methodological quality assessment (Chen et al. 2024). Previous overviews of acupuncture for PSD have primarily summarized the direction of treatment effects and the methodological quality of available reviews. The present study was designed to extend this perspective by integrating evidence mapping, AMSTAR 2 (A Measurement Tool to Assess Systematic Reviews 2) appraisal, outcome‐level Grading of Recommendations Assessment, Development and Evaluation (GRADE) assessment, primary‐study overlap analysis, and prescription‐pattern mining within a single framework. Rather than providing another pooled estimate of treatment efficacy, we aimed to characterize the structure, credibility, outcome coverage, redundancy, and intervention patterns of the existing evidence base, thereby identifying specific gaps relevant to future research and evidence synthesis. Accordingly, this study aimed to evaluate the methodological quality and certainty of the available evidence, assess primary‐study overlap and outcome coverage, and characterize recurrent acupuncture prescription patterns.

## 2 Methods

### 2.1 Study Design and Registration

This study was designed as an overview of SRs/MAs based on published SRs/MAs, with evidence‐map visualization and methodological quality assessment conducted at the review level. The overview was prospectively registered in PROSPERO on June 19, 2026 (CRD420261428106) and was reported in accordance with the PRISMA 2020 statement. Because this study synthesized evidence from previously published SRs/MAs and did not involve new human participants, animal experiments, or individual patient data, ethics approval was not required.

We aimed to characterize the structure of secondary evidence; therefore, effect estimates from primary trials were not re‐pooled, and no new meta‐analysis was performed. The original randomized controlled trials (RCTs) included in the reviews were extracted solely for overlap analysis and subsequent prescription pattern mining. Evidence‐map visualization followed the GEM methodological framework and incorporated the multidimensional presentation approach proposed by Ballesteros et al. (2017). The literature selection process was reported using a PRISMA flow diagram. The complete search strategies, final search date, and database‐specific retrieval counts are provided in Table S1.

### 2.2 Data Sources and Search Strategy

PubMed, Embase, the Cochrane Library, Web of Science, China National Knowledge Infrastructure (CNKI), the Chinese Biomedical Literature Database (CBM), VIP, and Wanfang were systematically searched from database inception to June 12, 2026. The search strategy combined controlled vocabulary terms and free‐text terms related to stroke, dysphagia, acupuncture, and SRs/MAs, and was adapted to the syntax of each database. No language restrictions were applied. Gray literature was searched through the OpenGrey online database, with the search restricted to records in English or Chinese.

### 2.3 Eligibility Criteria

Eligibility criteria were defined according to the PICOS framework:

*Population (P)*: Patients clinically diagnosed with PSD (Schaefer et al. 2024).

*Intervention (I)*: Acupuncture therapies, including filiform‐needle acupuncture, electroacupuncture, scalp acupuncture, tongue acupuncture, and eye acupuncture. To ensure intervention consistency, reviews evaluating non‐needle‐based stimulation modalities (e.g., laser acupuncture, transcutaneous electrical acupoint stimulation, transcutaneous electrical nerve stimulation) were excluded. Interventions involving explicit thermal stimulation (e.g., warm‐needle acupuncture, heat‐sensitive moxibustion, and other moxibustion‐related therapies) were also excluded to avoid potential confounding in attributing treatment effects. If a review included both acupuncture and non‐acupuncture stimulation interventions, and acupuncture‐specific results could not be separately extracted at the review level, the review was excluded. These exclusions applied only to the intervention arm; sham acupuncture was permitted as a comparator.

*Comparator (C)*: Conventional rehabilitation, pharmacotherapy, sham acupuncture, no treatment, or comparisons between different acupuncture regimens. For evidence‐structure classification, conventional rehabilitation, pharmacotherapy, sham acupuncture, and no treatment were categorized as non‐acupuncture comparators.

*Outcomes (O)*: Outcomes related to swallowing function, aspiration risk, complications, and safety, without restriction on specific scales or assessment methods.

*Study design (S)*: SRs/MAs in which the primary studies were RCTs.

Studies were excluded if any of the following criteria were met:

Publications that were not original SRs/MAs of primary studies, such as umbrella reviews, overviews, network meta‐analyses, methodological articles, animal studies, conference abstracts, editorials, expert opinions, or other non‐peer‐reviewed full‐text articles.

Studies in which the intervention did not meet the eligibility criteria, or in which it remained unclear after reviewing the full text and the Supporting Information as to whether the intervention qualified as acupuncture.

Studies from which PSD‐specific outcome data could not be separately extracted, or for which the full text remained unavailable after contacting the authors.

Duplicate publications or updated versions addressing the same topic, in which case only the most complete or most recent version was retained.

### 2.4 Study Selection and Data Processing

All the retrieved records were imported into EndNote X9 for management and de‐duplication. Two reviewers independently screened titles, abstracts, and full texts. Disagreements were resolved through discussion, and unresolved discrepancies were arbitrated by a third reviewer. The selection process is illustrated in the following flow diagram. Data were extracted using a standardized extraction form. The extracted items included the basic characteristics of the reviews, AMSTAR 2 assessment results, evidence indicators, and information on the primary trials identified from the reviews. The extracted review‐level characteristics are presented in Table S4, item‐level AMSTAR 2 assessments in Table S6A, outcome‐level evidence data in Table S7A–F, and primary‐trial correspondence data in Table S2.

All RCTs listed in the included SRs/MAs were extracted, and the full text was obtained whenever possible. For potentially duplicate publications, records were cross‐checked for title, authors, publication year, sample size, intervention, and comparator. Records that reported the same primary trial were merged, including duplicate publications in the same language and in both Chinese and English. After merging, each original RCT was retained as a single record in order to generate a de‐duplicated set of unique RCTs. This dataset was subsequently used for overlap analysis and acupuncture prescription pattern mining.

### 2.5 Methodological Quality Assessment

The methodological quality of the included SRs/MAs was assessed using AMSTAR 2, a tool with established validity and reliability for evaluating SRs/MAs (Shea et al. 2017). To evaluate the influence of Item 7 classification on the overall confidence ratings, two AMSTAR 2 configurations were assessed. The original AMSTAR 2 critical‐domain configuration was used as the reference assessment, while an alternative configuration in which Item 7 (provision of a list of excluded studies and reasons for exclusion) was not considered a critical domain was additionally evaluated. All other critical domains were assessed according to the original AMSTAR 2 guidance (Shea et al. 2017).

Overall confidence was classified according to AMSTAR 2 criteria: (Shea et al. 2017) high confidence (no critical weakness and no more than one noncritical weakness); moderate confidence (no critical weakness and more than one noncritical weakness); low confidence (one critical weakness); and critically low confidence (two or more critical weaknesses).

### 2.6 GRADE Assessment of the Certainty of Evidence

The certainty of evidence for all eligible outcomes reported in the included reviews was assessed using the GRADE approach. Each outcome was used as a unit of assessment, and evidence certainty was rated by considering five domains: risk of bias, inconsistency, indirectness, imprecision, and publication bias. As the included evidence was derived primarily from RCTs, the initial certainty was rated high and then downgraded as appropriate. The final certainty of evidence was categorized as high, moderate, low, or very low, with higher ratings indicating greater confidence in the evidence (Atkins et al. 2004).

Given the potential variation across the included SRs/MAs in the completeness and stringency of their GRADE assessments, the GRADE ratings reported in the original reviews were not adopted directly. Instead, all eligible outcome‐level evidence was independently reassessed by two reviewers using the same predefined GRADE criteria to enhance comparability across reviews. Downgrading decisions considered five domains: risk of bias, inconsistency, indirectness, imprecision, and publication bias, with one‐ or two‐level downgrading applied according to the severity of the concern. Detailed operational criteria are provided in Table S7G, and the complete outcome‐level GRADE assessments illustrating the application of these criteria are presented in Table S7A–F. Inter‐rater agreement was evaluated using the reviewers' independent assessments before consensus. Disagreements were subsequently resolved through discussion based on the predefined criteria, and the consensus ratings were used in the final analyses. Because the GRADE certainty categories are ordinal, agreement for the overall certainty rating was quantified using linearly weighted Cohen's kappa, together with the exact percentage agreement.

### 2.7 Classification of Conclusion Direction

Conclusions were classified using the five‐category framework proposed by Ballesteros et al. (2017), based on the authors' summary statements regarding the main research question in the abstract or conclusion sections of each included SR/MA. The classification criteria were as follows: A conclusion was deemed “beneficial” if authors reported clear benefits without major evidence limitations; “probably beneficial” if findings suggested a trend toward benefit but a definitive conclusion was withheld due to limitations or uncertainty; “inconclusive” if conclusions were inconsistent across studies or reviews or if methodological limitations precluded judgment; “no differential effect” if authors found no apparent difference between the intervention and comparator; and “harmful” if authors reported harmful effects or an increased risk of adverse outcomes.

### 2.8 Presentation and Visualization of the Evidence Structure

Consistent with the methodological framework of GEM, a multidimensional bubble plot was used to present the evidence distribution of the included reviews (Bragge et al. 2011). The x‐axis represented the direction of the review conclusions, and the y‐axis represented AMSTAR 2 methodological quality ratings. Bubble size was proportional to the number of original RCTs included in each review; bubble color indicated the main comparator type; and each bubble represented a single SR/MA.

To depict the evidence distribution across acupuncture modalities, comparator types, and outcome domains, a Sankey diagram was also generated. The unit of analysis in the Sankey diagram was the evidence link, defined as the pairing between one SR/MA and one outcome domain. When a single SR/MA reported multiple outcome domains, multiple evidence links were generated. The width of each flow was proportional to the number of evidence links corresponding to a given combination of acupuncture modality, comparator type, and outcome domain.

### 2.9 Extraction and De‐Duplication of Primary Data and CCA Calculation

To conduct overlap analysis and prescription pattern mining, we extracted primary RCTs from the eligible SRs/MAs. All primary trials used for prescription pattern analysis were derived from the original studies included in these SRs/MAs. This approach allowed us to identify acupuncture prescription patterns frequently incorporated and reported within the current body of secondary evidence. All RCT records were then collated from the reference lists of the included SRs/MAs. Records with insufficient information to determine whether they referred to the same trial were conservatively retained as separate records at this stage and rechecked after full‐text retrieval.

A two‐stage matching process was applied to identify potential duplicate publications of the same trial or duplicate Chinese–English publications. Duplicates were first merged within the same language, followed by cross‐language matching using title, author information, publication year, sample size, intervention, and comparator characteristics. This process yielded a set of unique RCTs. Acupuncture prescription elements were then extracted from these unique RCTs with available full texts for subsequent pattern mining. No further risk‐of‐bias assessment or certainty‐of‐evidence appraisal was conducted for these primary trials. Cross‐language matching and the final correspondence among unique trials are detailed in Table S2.

To quantify the extent of overlap in primary studies across the included SRs/MAs, we performed an overlap analysis using the corrected covered area (CCA). First, all RCTs included in each SR/MA were compiled to create the overlap dataset. The CCA was calculated according to the method proposed by Pieper et al. (2014):

$$CCA\;=\frac{N\;-\;c}{c\;\times \;(r\;-\;1)}$$

where N denotes the total number of citation occurrences in the dataset, r denotes the number of included SRs/MAs, and c denotes the number of unique RCTs after cross‐language de‐duplication. CCA values were interpreted as follows: 0%–5%, slight overlap; 6%–10%, moderate overlap; 11%–15%, high overlap; and > 15%, very high overlap.

### 2.10 Prescription Data Extraction and Association Rule Mining

The RCTs used for prescription pattern mining were derived from the primary studies included in the eligible SRs/MAs; thus, the findings reflect acupuncture prescription characteristics incorporated and reported within the current body of secondary evidence. Association rule mining was performed using the Apriori algorithm, with each individual prescription treated as one unit of analysis (Agrawal and Srikant 1994).

To avoid duplicate reporting of reciprocal one‐item rules (e.g., A→B and B→A), a redundancy‐reduction procedure was applied. For each unordered item pair, rules were ranked sequentially by confidence, support, and lift, and only the highest‐ranking rule was retained for presentation. Lift was used to indicate the strength of association, with a lift value greater than one suggesting a positive association; however, lift was not used as a threshold for rule selection in this study.

Support represents the proportion of prescriptions containing both the antecedent and consequent, whereas confidence represents the proportion of prescriptions containing the antecedent that also contain the consequent. Lift compares the observed co‐occurrence with that expected under independence, with values > 1 indicating a positive association. Because item frequencies and rule densities differed across the four analytical dimensions, dimension‐specific minimum support thresholds were selected after sensitivity testing to balance retention of recurrent patterns against excessive low‐frequency rules. The primary minimum support thresholds were 10% for acupoints, 15% for meridians, 20% for stimulation sites, and 15% for specific acupoint attributes, with a minimum confidence threshold of 80% across all four dimensions.

To examine the robustness of parameter selection, we conducted threshold sensitivity analyses using multiple alternative parameter settings and determined dimension‐specific minimum support thresholds. The consistency of core association rules across different parameter settings was compared to ensure that the final thresholds could reliably identify acupuncture prescription patterns with potential value for subsequent validation.

### 2.11 Network Visualization and Outcome‐Domain Coverage Statistics

To characterize the prescription features of RCTs derived from the secondary evidence base, each acupuncture prescription was treated as the unit of analysis. Co‐occurrence patterns of acupoints, meridians, stimulation sites, and specific acupoint attributes within the same prescription were summarized and used to construct co‐occurrence networks. In these networks, nodes represented acupoints, meridians, stimulation sites, or specific acupoint attributes, with node size proportional to their frequency across all prescriptions. Edges represented the co‐occurrence of two items within the same prescription, and edge width was proportional to co‐occurrence strength. To improve readability, only the top 10–20 most frequent nodes were displayed for each dimension, and minimum co‐occurrence thresholds were applied to control network density. The co‐occurrence networks were constructed solely for descriptive visualization and were not used to infer treatment effects.

Outcome‐domain coverage was summarized using the included SRs/MAs as the unit of analysis by counting the number of reviews that reported each outcome domain. Because a single review could report multiple outcome domains, non‐mutually exclusive counting was applied; therefore, the summed number of reviews across outcome domains could exceed 45. A further distinction was made between “any‐outcome” and “primary‐outcome” coverage. Any‐outcome coverage was defined as an outcome domain reported either as a primary or secondary outcome in a review, whereas primary‐outcome coverage was counted only when the review explicitly defined that outcome domain as a primary outcome.

### 2.12 Software and Figure Generation

Data analyses and figure generation were performed using Microsoft Excel and R software version 4.3.3. Evidence distributions were visualized using bubble plots, heat maps, and related graphical approaches. The R code used for association rule mining, threshold sensitivity analysis, and graphical visualization is provided as Code S1.

## 3 Results

### 3.1 Literature Search and Study Selection

The systematic search of Chinese and English databases identified 310 records. After de‐duplication, 174 records remained for title and abstract screening. Subsequently, 121 full‐text articles were retrieved and assessed, of which 45 SRs/MAs were ultimately included. During full‐text assessment, 76 articles were excluded; 24 of these were excluded because their intervention involved non‐acupuncture stimulation. The study selection process is shown in Figure 1, and reasons for exclusion are provided in Table S3.

**FIGURE 1:**
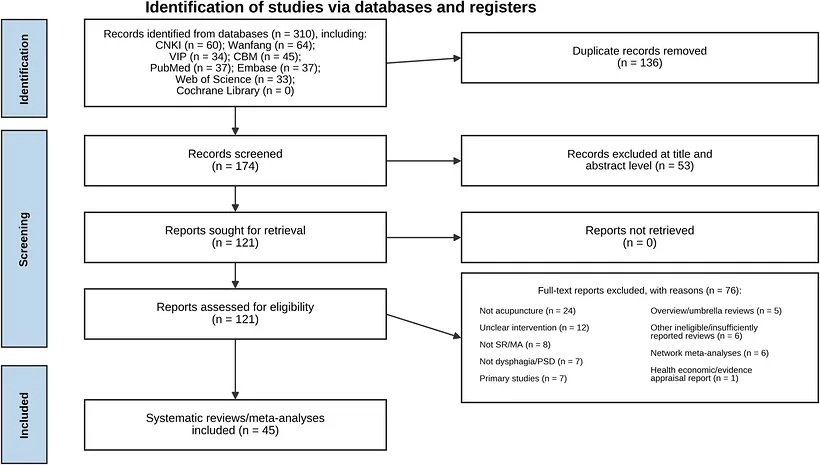
PRISMA flow diagram of the study selection process.

### 3.2 Characteristics of the Included Reviews and Overlap Analysis

The 45 included SRs/MAs were published between 2006 and 2025. Their basic characteristics are presented in Table S4. In terms of publication year, 17 reviews (37.8% of the included studies) were published between 2006 and 2019, while a larger proportion, 28 reviews (62.2%), were published between 2020 and 2025.

A total of 894 primary RCT records were extracted. After de‐duplication and merging, 779 unique RCTs were identified. According to the method proposed by Pieper et al. (2014), the CCA was calculated as 0.34% (Table S5), indicating slight overlap. As full texts were unavailable for some unique RCTs, prescription pattern mining was limited to 470 unique RCTs with accessible full texts, from which 512 acupuncture prescriptions were extracted.

### 3.3 Evidence Flows

Evidence links primarily involved manual acupuncture (*n* = 80) and adjuvant therapy comparisons (*n* = 62), as shown in Figure S1. Overall, adjuvant therapy represented the dominant comparator design, whereas direct comparative acupuncture evidence was relatively limited. At the outcome‐domain level, evidence links primarily covered total effective rate (*n* = 45) and swallowing function scales (*n* = 39), while coverage of key patient‐important outcomes, such as aspiration/pneumonia (*n* = 5) and safety and adverse events (*n* = 6) remained limited.

### 3.4 Study Characteristics

Manual acupuncture was the most common intervention category among the included reviews, representing 62.2% (28/45). Regarding comparator design, adjuvant therapy was the most frequent category (21/45, 46.7%), followed by non‐acupuncture controls (12/45, 26.7%; including conventional rehabilitation, pharmacotherapy, sham acupuncture, or no treatment), comparative acupuncture (7/45, 15.6%), and mixed controls (5/45, 11.1%). The corresponding distributions, including those of co‐interventions, are shown in Figure S2.

### 3.5 Methodological Quality Assessment

Under the original AMSTAR 2 critical‐domain configuration, which was retained as the reference assessment, one review (2.2%) was rated as moderate quality, eight (17.8%) as low quality, and 36 (80.0%) as critically low quality; none were rated as high quality. Detailed item‐level results are presented in Figure S3 and Table S6A. Under the alternative configuration in which Item 7 was not considered a critical domain, the distribution changed to five moderate‐quality reviews, 30 low‐quality reviews, and 10 critically low‐quality reviews. Comparison of the two configurations therefore showed that the classification of Item 7 substantially influenced the overall confidence ratings (Table S6B,C).

The main methodological weaknesses stemmed from three key domains (Table S6A): (1) inadequate prospective protocol registration (Item 2), as only 15.6% (7/45) reported prior protocol registration, such as registration in the International Prospective Register of Systematic Reviews (PROSPERO); (2) insufficient transparency regarding excluded studies (Item 7), with only 8.9% (4/45) providing a complete list of excluded studies and their reasons; and (3) poor reporting of funding sources for primary RCTs (Item 10), as only 2.2% (1/45) reported the funding sources of the original trials. In contrast, compliance was relatively high for Item 1, which assesses whether the research question and inclusion criteria were clearly defined, and Item 5, which assesses whether study selection was performed independently by two reviewers.

### 3.6 GRADE Assessment of the Certainty of Evidence

Before consensus, exact agreement between the two reviewers for the overall GRADE certainty rating was 68.5% (111/162), with a linearly weighted Cohen's *κ* of 0.456. Detailed agreement statistics are provided in Table S7H.

A total of 162 outcome‐level entries were assessed using the GRADE approach. Overall, the certainty of evidence was predominantly low or very low: low certainty (92 entries, 56.8%), very low certainty (66 entries, 40.7%), and moderate certainty (four entries, 2.5%). No high‐certainty evidence was identified.

By outcome domain, total effective rate and related composite outcomes constituted the largest proportion of entries (62, 38.3%), followed by swallowing function scales (60, 37.0%) and instrumental swallowing assessments (19, 11.7%). Outcomes related to quality of life and functional recovery (14, 8.6%), aspiration/pneumonia (4, 2.5%), and safety/adverse events (3, 1.9%) were comparatively underrepresented. Detailed results are provided in Table S7A–F.

### 3.7 Direction of Conclusions and Methodological Quality

Figure 2 shows that the conclusions of the included SRs/MAs were predominantly classified as “probably beneficial.” Overall, 44 of 45 reviews (97.8%) were classified as “probably beneficial,” whereas one review (2.2%) was classified as “inconclusive.” Notably, several reviews that included a relatively large number of RCTs were still rated as having low or critically low methodological quality.

**FIGURE 2:**
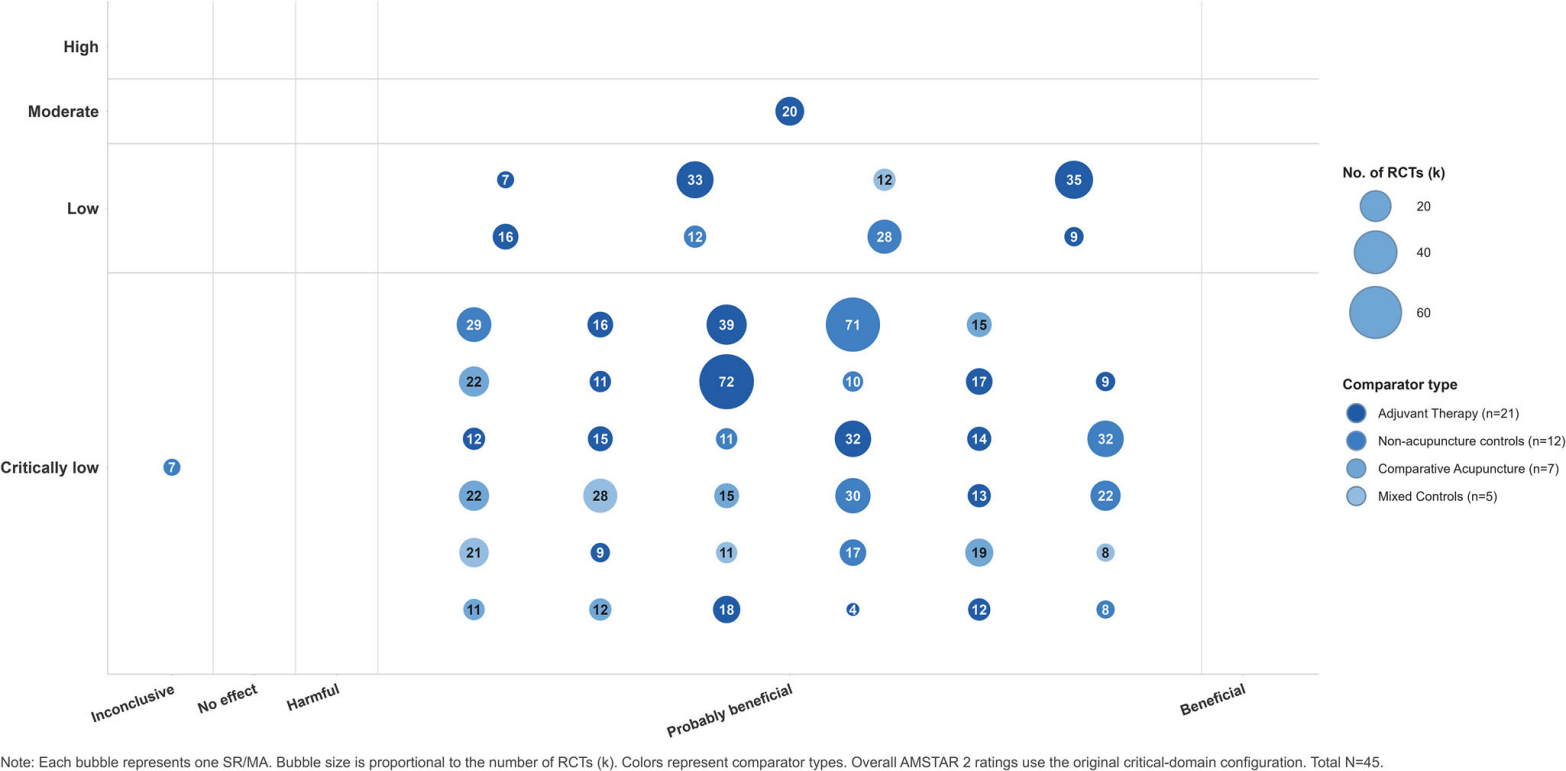
Evidence bubble plot of acupuncture for the treatment of poststroke dysphagia.

### 3.8 Hierarchical Distribution of Outcome Domains

Outcome‐domain coverage was markedly uneven (Figure 3). Among the 45 included SRs/MAs, the total effective rate was the most frequently reported outcome domain (45 reviews, 100.0%), followed by swallowing function scales (39 reviews, 86.7%) and instrumental swallowing assessments (24 reviews, 53.3%). However, these outcomes were not commonly specified as primary outcomes, being designated as such in only 16, 10, and 17 reviews, respectively.

**FIGURE 3:**
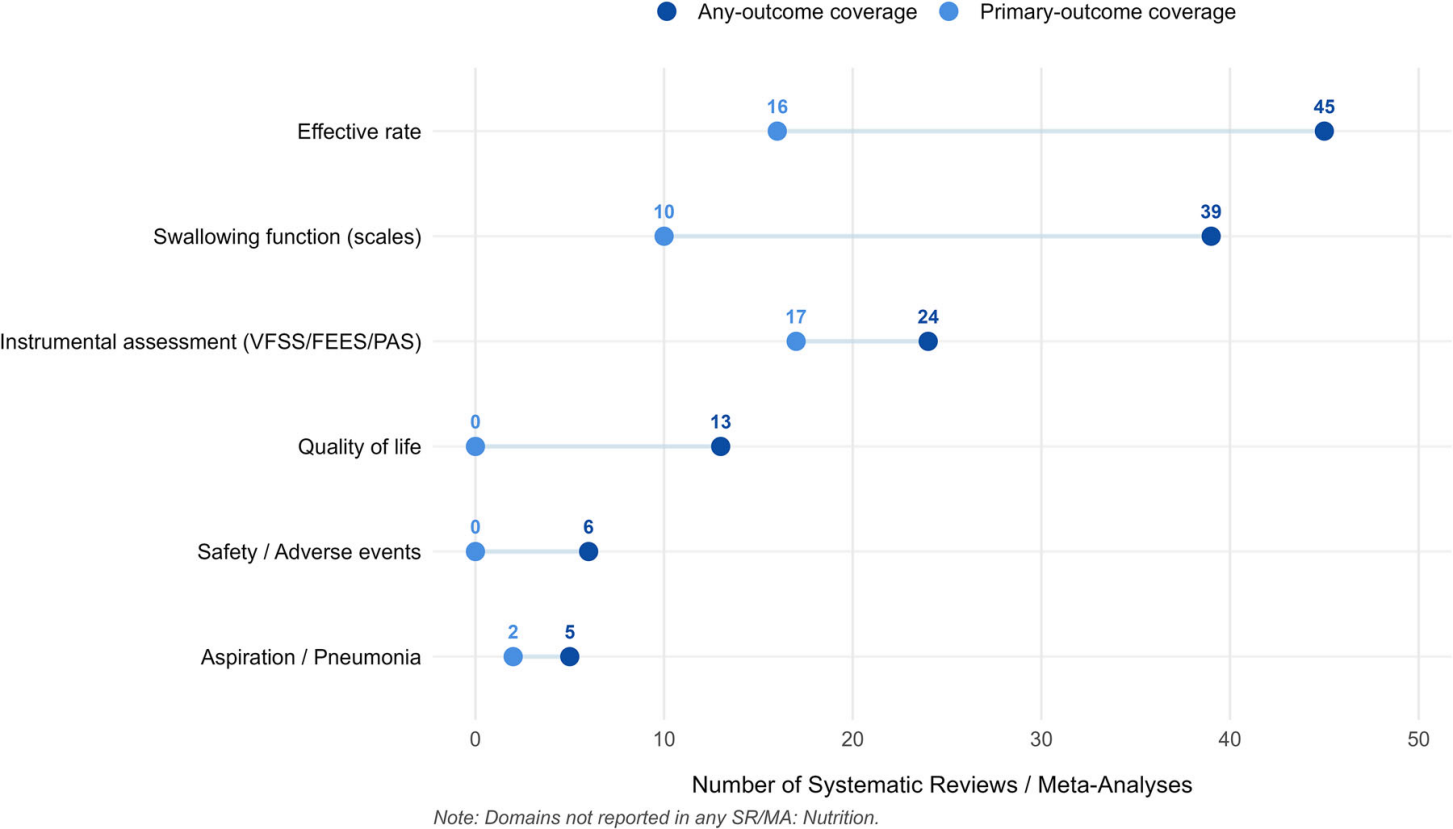
Distribution of outcome‐domain coverage.

**FIGURE 4:**
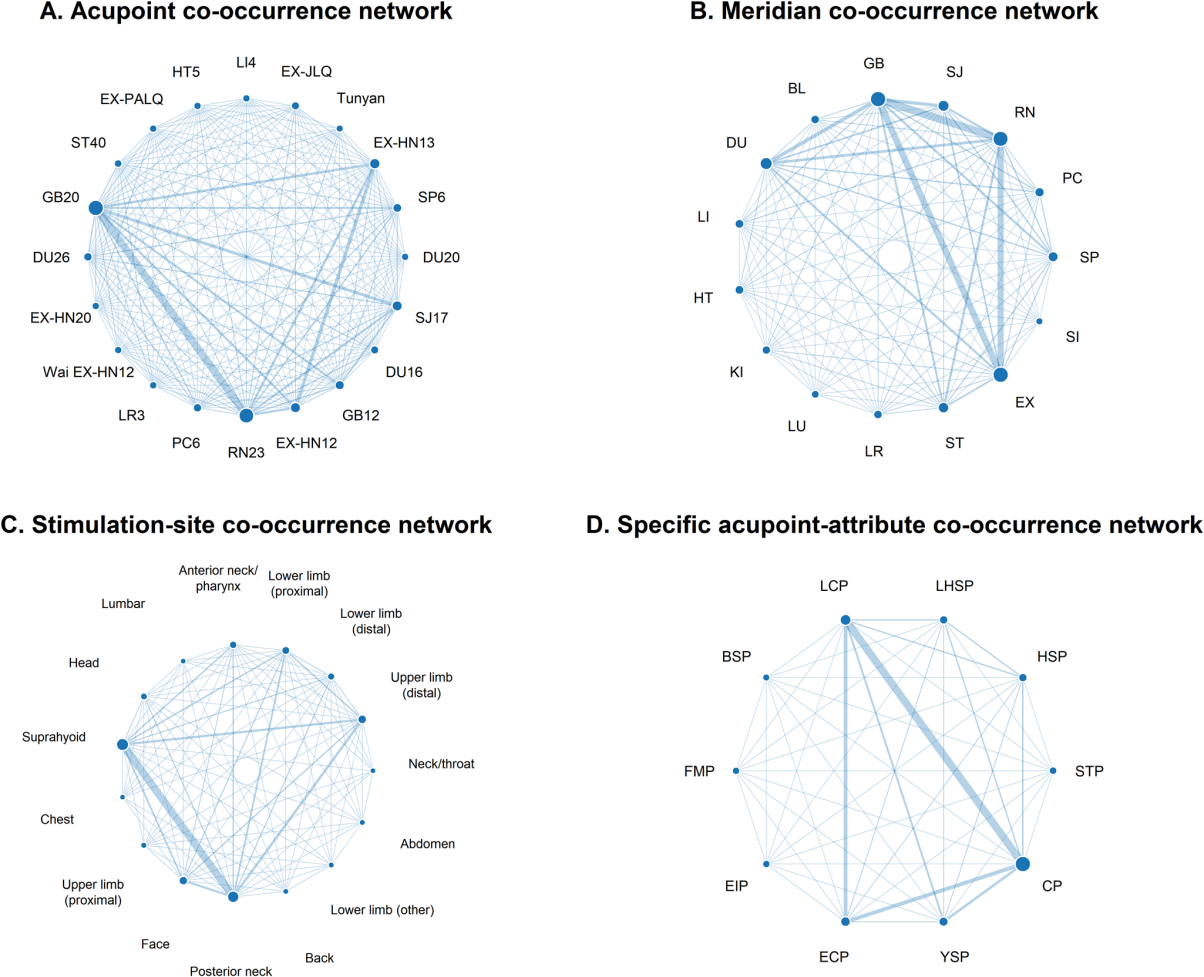
Prescription co‐occurrence networks. (A) Acupoint co‐occurrence network showing the top 20 high‐frequency acupoints: GB20, Fengchi; RN23, Lianquan; EX‐HN13, Yuye; EX‐HN12, Jinjin; SJ17, Yifeng; GB12, Wangu; SP6, Sanyinjiao; PC6, Neiguan; DU26, Shuigou/Renzhong; DU16, Fengfu; EX‐JLQ, Jia‐Lianquan; ST40, Fenglong; EX‐HN20, Shang‐Lianquan; LI4, Hegu; EX‐PALQ, Pang‐Lianquan; LR3, Taichong; Wai EX‐HN12, Wai‐Jinjin; DU20, Baihui; HT5, Tongli; and Tunyan, swallowing acupoint. (B) Meridian co‐occurrence network showing the top 15 meridian or attribution categories: BL, Bladder Meridian of Foot‐Taiyang; DU, Governor Vessel; EX, extraordinary acupoints; GB, Gallbladder Meridian of Foot‐Shaoyang; HT, Heart Meridian of Hand‐Shaoyin; KI, Kidney Meridian of Foot‐Shaoyin; LI, Large Intestine Meridian of Hand‐Yangming; LR, Liver Meridian of Foot‐Jueyin; LU, Lung Meridian of Hand‐Taiyin; PC, Pericardium Meridian of Hand‐Jueyin; RN, Conception Vessel; SI, Small Intestine Meridian of Hand‐Taiyang; SJ, Sanjiao Meridian of Hand‐Shaoyang; SP, Spleen Meridian of Foot‐Taiyin; ST, Stomach Meridian of Foot‐Yangming. (C) Stimulation‐site co‐occurrence network showing the top 15 stimulation sites: suprahyoid region, posterior neck, anterior neck/pharynx, face, distal upper limb, proximal upper limb, distal lower limb, proximal lower limb, chest, back, head, abdomen, lumbar region, neck/throat, and other lower‐limb sites. (D) Specific acupoint‐attribute co‐occurrence network showing the top 10 categories: BSP, back‐shu points; CP, crossing points; ECP, eight confluent points; EIP, eight influential points; FMP, front‐mu points; HSP, He‐sea points; LCP, Luo‐connecting points; LHSP, lower He‐sea points; STP, five‐shu transport points; YSP, Yuan‐source points.

Quality‐of‐life outcomes were reported in 13 reviews (28.9%), but none designated them as primary outcomes. Aspiration/pneumonia and safety/adverse events were infrequently reported, appearing in five reviews (11.1%) and six reviews (13.3%), respectively; however, only two reviews specified aspiration/pneumonia as a primary outcome. Nutrition‐related outcomes were not reported in any of the included reviews (0/45). The operational definitions, commonly used assessment tools, and detailed coverage of each outcome domain are provided in Table S8.

### 3.9 Core Acupoints and Prescription Patterns

A total of 512 acupuncture prescriptions were analyzed. The Apriori algorithm was used to identify within‐prescription item co‐occurrence relationships, and circular co‐occurrence networks were then constructed to visualize these structures across four dimensions: acupoints, meridians, stimulation sites, and specific acupoint attributes (Figure 4). Based on threshold sensitivity analyses, the minimum support thresholds were set as follows: ≥ 10% for the acupoint dimension, ≥ 15% for the meridian dimension, ≥ 20% for the stimulation‐site dimension, and ≥ 15% for the specific acupoint‐attribute dimension. A minimum confidence threshold of ≥ 80% was uniformly applied across all dimensions.

Under alternative parameter settings, retention of the primary top‐10 rules decreased mainly when the confidence threshold was increased to 90%, whereas most rules were retained under changes in support thresholds or the additional lift > 1 criterion (Table S9).

The most frequently used acupoints included Fengchi (GB20), Lianquan (RN23), Jinjin (EX‐HN12), and Yuye (EX‐HN13). The principal meridian categories were the Sanjiao Meridian of Hand‐Shaoyang (SJ), Gallbladder Meridian of Foot‐Shaoyang (GB), Stomach Meridian of Foot‐Yangming (ST), and Conception Vessel (RN). Principal stimulation sites were concentrated in the posterior neck, suprahyoid region, and distal upper limb. Commonly used specific acupoint attributes included crossing points (CP), Luo‐connecting points (LCP), and eight confluent points (ECP). Their corresponding frequency distributions are shown in Figure S4.

Table 1 presents the core association networks of acupuncture prescriptions for PSD. The most common acupoint combinations identified were EX‐HN12 → EX‐HN13, GB20 + EX‐HN12 → EX‐HN13, and GB20 + EX‐HN13 → EX‐HN12. In the meridian dimension, the combinations with the highest support were SJ → GB, ST → RN, and SP → GB. For the stimulation‐site dimension, the top three core associations were posterior neck → suprahyoid, upper limb (distal) → posterior neck, and face → posterior neck. In the specific acupoint‐attribute dimension, the combinations with the highest support were LCP → CP, ECP → CP, and ECP → LCP. Table S9 provides the results of the threshold sensitivity analyses, indicating that the core rules identified under different parameter settings were generally consistent.

**TABLE 1:** Top 10 association rules by dimension for acupuncture prescriptions in poststroke dysphagia.

Dimension	Rule (antecedent → consequent)	Cases	Support (%)	Confidence (%)	Lift
Acupoints	EX‐HN12 → EX‐HN13	156	30.47	98.73	3.08
	SJ17 → GB20	142	27.73	90.45	1.37
	GB20 + EX‐HN12 → EX‐HN13	122	23.83	98.39	3.07
	GB20 + EX‐HN13 → EX‐HN12	122	23.83	94.57	3.06
	RN23 + EX‐HN12 → EX‐HN13	120	23.44	99.17	3.10
	RN23 + EX‐HN13 → EX‐HN12	120	23.44	95.24	3.09
	GB12 → GB20	119	23.24	94.44	1.43
	RN23 + EX‐HN13 → GB20	105	20.51	83.33	1.27
	GB20 + EX‐HN13 → RN23	105	20.51	81.40	1.29
	RN23 + EX‐HN12 → GB20	101	19.73	83.47	1.27
Meridians	SJ → GB	160	31.25	93.57	1.37
	ST → RN	123	24.02	80.92	1.23
	SP → GB	116	22.66	87.88	1.29
	RN + DU → GB	116	22.66	80.56	1.18
	RN + SJ → GB	113	22.07	92.62	1.36
	EX + DU → GB	102	19.92	83.61	1.23
	GB + ST → RN	99	19.34	83.19	1.26
	RN + ST → GB	99	19.34	80.49	1.18
	DU + SJ → GB	95	18.55	91.35	1.34
	EX + SJ → GB	89	17.38	95.70	1.40
Stimulation sites	Posterior neck → suprahyoid	332	64.84	86.01	1.04
	Upper limb (distal) → posterior neck	168	32.81	86.15	1.14
	Face → posterior neck	165	32.23	90.16	1.20
	Upper limb (distal) → suprahyoid	162	31.64	83.08	1.01
	Lower limb (proximal) → posterior neck	140	27.34	88.61	1.18
	Suprahyoid + upper limb (distal) → posterior neck	140	27.34	86.42	1.15
	Posterior neck + upper limb (distal) → suprahyoid	140	27.34	83.33	1.01
	Face + suprahyoid → posterior neck	132	25.78	92.96	1.23
	Posterior neck + face → suprahyoid	132	25.78	80.00	0.97
	Lower limb (proximal) → suprahyoid	127	24.80	80.38	0.97
Specific acupoint attributes	LCP → CP	204	39.84	99.51	1.12
	ECP → CP	138	26.95	99.28	1.12
	ECP → LCP	135	26.37	97.12	2.43
	ECP + LCP → CP	134	26.17	99.26	1.12
	CP + ECP → LCP	134	26.17	97.10	2.43
	YSP → CP	110	21.48	98.21	1.11
	YSP → LCP	94	18.36	83.93	2.10
	LCP + YSP → CP	93	18.16	98.94	1.12
	CP + YSP → LCP	93	18.16	84.55	2.11
	HSP → CP	79	15.43	100.00	1.13

In the acupoint network (Figure 4A), GB20 and RN23 occupied central positions and exhibited relatively high connectivity. High‐support co‐occurrence relationships were mainly concentrated in local and adjacent acupoint combinations, such as GB20 with Yifeng and RN23 with EX‐HN12 and EX‐HN13. The meridian network (Figure 4B) revealed that the Gallbladder Meridian of Foot‐Shaoyang and the Conception Vessel were frequently observed, consistent with the meridian attribution of the high‐frequency acupoints. In the stimulation‐site network (Figure 4C), the suprahyoid region and posterior neck were the most frequently targeted stimulation areas. The specific acupoint‐attribute network (Figure 4D) showed that CPs accounted for the largest proportion.

## 4 Discussion

The main contribution of this overview lies not in reiterating that the existing acupuncture evidence is methodologically limited, but in characterizing how those limitations are distributed across the current evidence base. By integrating methodological appraisal, outcome‐level certainty assessment, primary‐study overlap analysis, outcome‐domain mapping, and prescription‐pattern mining, this study provides a multidimensional view of the structure and credibility of the secondary evidence on acupuncture for PSD. The findings therefore extend beyond a summary of treatment direction and help identify where evidence is concentrated, where clinically important outcomes remain underrepresented, and which prescription patterns warrant further prospective validation.

### 4.1 Methodological Quality, Overlap, and Certainty of Evidence

The CCA calculated in this study was 0.34%, which indicates a slight overlap according to the criteria proposed by Pieper et al. (2014). It should be noted that a slight overlap merely indicates limited cross‐inclusion of primary trials among different SRs/MAs, rather than implying the establishment of a sufficiently coherent evidence synthesis framework in this field. Methodological work by Lunny et al. (2021) on managing overlap across reviews emphasizes that the overlap of primary studies between systematic reviews is a key issue when synthesizing secondary evidence. Its interpretation, moreover, should not be detached from the review scope, eligibility criteria, and data‐processing procedures.

Given the present findings, the low CCA more likely reflects heterogeneity among the included SRs/MAs regarding their defined research questions, acupuncture modalities, comparator settings, co‐interventions, and outcome selection. This heterogeneity has resulted in a relatively dispersed set of primary RCTs across the reviews. These findings suggest that the current body of secondary evidence has not yet accumulated around a stable set of core research questions. However, low overlap should not be interpreted as direct evidence of strong evidence quality. The reliability of the conclusions must therefore be judged by separately examining the methodological quality of the SRs/MAs and the certainty of evidence at the outcome level. In this study, the overall methodological quality of the included SRs/MAs was low, with no review rated as high quality. This suggests that, although secondary evidence on acupuncture for PSD has continued to accumulate, methodological rigor has not improved in parallel.

Item‐level analysis using AMSTAR 2 showed that the main weaknesses were insufficient prospective protocol registration, inadequate reporting of excluded studies and reasons for exclusion, and poor disclosure of funding sources for primary RCTs. Insufficient protocol registration reflects limited prospective standardization at the design stage and may increase the risk of selective presentation of results. Inadequate reporting of excluded studies weakens the transparency and traceability of the study selection process. PRISMA 2020 emphasizes that systematic reviews should report methods and results completely and transparently to enhance the verifiability of the review process (Page et al. 2021). Kirkham et al. (2010) further showed that selective outcome reporting may influence systematic reviews' judgments of intervention effects. In addition, although insufficient reporting of funding information does not directly prove bias in study conclusions, it reduces the transparency of result interpretation and hinders the identification and discussion of potential funding‐related bias (Lundh et al. 2017). Taken together, the major problems in this field are insufficient prespecification of review protocols, limited process transparency, and incomplete interpretation of findings. The overall confidence ratings were highly sensitive to the classification of Item 7. Under the original AMSTAR 2 critical‐domain configuration, which was retained as the reference assessment, 36 of 45 reviews were rated as critically low. The alternative configuration produced fewer critically low ratings, indicating that Item 7 classification substantially influenced the overall methodological grading. Comparisons with other studies should therefore primarily refer to the original AMSTAR 2 configuration.

Beyond the methodological quality of the SRs/MAs, the certainty of evidence at the outcome level also warrants careful consideration. Accordingly, this study used the GRADE approach to uniformly reappraise the certainty of evidence for each outcome reported in the included SRs/MAs (Hultcrantz et al. 2017). The results showed that the certainty of the current evidence was predominantly low or very low, with no high‐certainty evidence identified. Although most SRs/MAs tended to conclude that acupuncture may be beneficial, the evidentiary basis supporting this judgment remains limited (Wang et al. 2022). These findings are broadly consistent with recent meta‐analyses and an overview of acupuncture for PSD, which also reported generally favorable findings alongside important limitations in methodological quality and certainty of evidence (Li et al. 2024; Wang et al. 2022; Xu et al. 2025). The present study extends this evidence by integrating outcome‐domain mapping and prescription‐pattern analysis with methodological appraisal and primary‐study overlap assessment. Further analysis indicated that risk of bias was the principal reason for downgrading evidence certainty, a finding consistent with previous reports on the application of GRADE in systematic reviews of traditional Chinese medicine. Nearly all outcome categories—including composite outcomes such as total effective rate, swallowing function scales, aspiration/pneumonia, and safety outcomes—were affected to varying degrees by risk of bias. This suggests that key methodological aspects of the primary RCTs, including randomization procedures, allocation concealment, and the implementation or reporting of blinding, remain insufficient.

The reasons for downgrading differed across outcome domains. Composite outcomes such as total effective rate were more susceptible to indirectness; swallowing function scale outcomes were more often affected by inconsistency, reflecting substantial heterogeneity across studies; and aspiration/pneumonia and safety outcomes were more frequently downgraded for imprecision because of the limited number of studies, small sample sizes, and unstable effect estimates (Guyatt et al. 2011). Overall, risk of bias remained the common reason for downgrading across most outcomes. Therefore, although most SRs/MAs reached broadly positive conclusions suggesting that acupuncture may be beneficial, this consistency primarily reflects a similar direction of conclusions rather than robust support based on high‐certainty evidence. Given the low methodological quality, low certainty of GRADE evidence, and predominance of total effective rate as an outcome measure observed in this study, the currently positive conclusions should be interpreted with caution.

From a neurological rehabilitation perspective, these findings should be interpreted with caution. Although acupuncture may be considered as a complementary intervention for PSD, the current evidence is limited by low methodological quality and low certainty. Therefore, acupuncture should not be presented as a definitive standalone treatment, but rather as a potential adjunctive option within multidisciplinary dysphagia rehabilitation when delivered by trained practitioners and combined with standard swallowing assessment and rehabilitation care.

### 4.2 Outcome Selection and Study Design

The generation of high‐quality evidence depends not only on methodological rigor in study conduct but also on the appropriateness of trial design and outcome selection. Taken together, the evidence map identified two principal gaps in the current literature: the underrepresentation of patient‐important outcomes, particularly nutritional status, aspiration/pneumonia, safety, and quality of life, and the limited direct comparative evidence between acupuncture and other active interventions. Indeed, the findings of this study indicate that current evidence on acupuncture for PSD is limited primarily by outcome selection, comparator design, and the clinical interpretability of study results. Specifically, outcome selection was markedly imbalanced, with most studies focusing on total effective rate, swallowing function scales, and instrumental swallowing assessments, while outcomes related to quality of life, aspiration/pneumonia, safety, and nutrition received insufficient attention. Among these outcomes, the total effective rate warrants particular caution. Although it was the most frequently reported outcome, it does not represent a standardized or stable clinical construct; rather, it is often derived through secondary aggregation of symptoms, scale scores, or investigator‐defined criteria. Consistent with this concern, previous systematic reviews of acupuncture have also regarded the total effective rate as an insufficiently validated, post hoc composite‐type outcome (Kwon et al. 2021).

For PSD, improvements in swallowing scores, reductions in aspiration, lower incidence of pneumonia, restoration of oral intake, and gains in quality of life are not clinically interchangeable. Consequently, summarizing these heterogeneous changes as a single positive result using the total effective rate may weaken the clinical interpretability of the findings. Notably, while the total effective rate is not identical to a composite outcome in the strict methodological sense, its practice of combining improvements across different clinical dimensions carries similar interpretive risks. Methodological studies of composite outcomes have likewise shown that combining outcomes with different clinical meanings may lead to misleading interpretations of treatment effects (Cordoba et al. 2010). Therefore, the total effective rate may be used as a supplementary descriptive measure but should not serve as the core basis for determining the true clinical benefit of acupuncture for PSD.

Further consideration of primary outcome designation indicates that current studies are affected not only by imbalanced outcome coverage but also by a clear outcome‐orientation bias. Indeed, existing research has focused predominantly on whether swallowing function improves, while giving insufficient attention to patient‐important outcomes such as aspiration, pneumonia, nutritional status, quality of life, and safety (Rjoob et al. 2022; Dziewas et al. 2021). This persistent prioritization of functional improvement indicators as primary outcomes, coupled with inadequate coverage of patient‐important outcomes, ultimately weakens the ability of the current evidence to support conclusions about meaningful clinical benefit (Kirkham et al. 2010). In particular, the complete absence of nutrition‐related outcomes in the included reviews is noteworthy. For patients with PSD, nutritional status not only influences the overall rehabilitation trajectory but is also closely associated with infection risk, physical recovery, and long‐term functional outcomes; it should therefore not remain peripheral in outcome evaluation frameworks (Wirth et al. 2013). Moreover, this lack of standardization in outcome frameworks is consistent with the low overlap observed in this study, suggesting substantial variation across the existing body of secondary evidence in the definition of research questions, eligibility criteria, and outcome selection.

In terms of outcome measurement, subjective scales, clinical severity ratings, and instrumental swallowing assessments should be regarded as complementary rather than interchangeable. Swallowing function scales, severity ratings, and swallowing‐related quality‐of‐life questionnaires can capture patients' perceived dysphagia burden, degree of functional limitation, and perceived treatment benefit. For instance, instruments such as the SWAL‐QOL can be used to assess the impact of dysphagia on quality of life (McHorney et al. 2002). Similarly, the Dysphagia Severity Rating Scale (DSRS) reflects the functional status of patients with PSD in real‐world eating and feeding management by incorporating liquid intake, dietary texture modification, and the need for supervision during feeding. In their study, Everton et al. (2020) validated the measurement properties of the DSRS in stroke patients, showing that it has good validity, reliability, and responsiveness, with a reported minimal clinically important difference of approximately one point. The DSRS can therefore be used to describe dysphagia severity and to determine whether changes in scores during follow‐up are clinically meaningful. Nevertheless, despite their utility, subjective scales and clinical severity ratings should not be viewed as substitutes for instrumental swallowing assessments. Instead, they remain particularly valuable in long‐term follow‐up and real‐world clinical settings. For patients who cannot repeatedly undergo examinations such as fiberoptic endoscopic evaluation of swallowing (FEES) or videofluoroscopic swallowing studies, these scales provide an important supplementary means of continuously monitoring functional changes.

Future studies should incorporate swallowing function, aspiration risk, nutritional status, quality of life, safety, and long‐term follow‐up outcomes within a core outcome set (COS) framework, complete with standardized definitions, measurement instruments, and reporting methods for each outcome (Kirkham et al. 2016). However, while instrumental swallowing assessments were more frequently designated as primary outcomes, their overall coverage has remained limited. Consequently, they have not yet been integrated into a comprehensive evaluation framework alongside aspiration/pneumonia, nutritional status, quality of life, and safety outcomes.

The 2021 guideline jointly issued by the European Stroke Organisation and the European Society for Swallowing Disorders emphasizes that instrumental assessment provides an important basis for identifying aspiration and developing targeted rehabilitation strategies (Dziewas et al. 2021). Langmore (2017) also demonstrated the important role of FEES in the assessment and management of pharyngeal‐phase dysphagia, as it can provide more direct, objective information for evaluating swallowing function. In addition, the Penetration–Aspiration Scale, a classic grading tool for penetration and aspiration, supports standardized interpretation of instrumental assessment findings (Rosenbek et al. 1996). Therefore, future studies should further incorporate objective examinations such as FEES or videofluoroscopic swallowing studies into standardized assessment systems, while also improving the standardized reporting of subjective outcomes. This would facilitate the integration of subjective assessment with objective instrumental evaluation and improve the comparability of treatment‐effect estimates. Objective instrumental assessment is crucial not only for determining whether swallowing function has improved but also for accurately identifying aspiration. Moreover, without standardized instrumental assessment, the interpretation of key clinical outcomes, such as pneumonia, may also be limited (Dziewas et al. 2021; Langmore 2017; Rosenbek et al. 1996).

Beyond outcome selection, the characteristics of evidence flows and comparator designs also affect the interpretation of existing findings. This study showed that manual acupuncture was the predominant intervention category, while adjuvant therapy was the most common comparator category, typically involving acupuncture added to usual care or rehabilitation. This suggests that existing studies have primarily examined the incremental benefit of acupuncture as an adjunctive therapy, whereas direct comparative evidence between acupuncture and other active treatments remains limited. Although such designs more closely resemble clinical practice, they also make it more difficult to isolate the specific effects of acupuncture (Zhang et al. 2022; Lim et al. 2024).

From a rehabilitation perspective, add‐on intervention designs are more consistent with the real‐world use of acupuncture, as acupuncture is typically delivered alongside swallowing training, rehabilitation care, or pharmacotherapy in clinical settings. The key issue, therefore, is whether the content, intensity, and implementation of concomitant treatments are clearly specified and fully reported. If the objective is to evaluate the specific effect of acupuncture, interference from co‐interventions should be minimized as far as possible, sham acupuncture should be used as a comparator where feasible, and allocation concealment, blinding of outcome assessors, and blinding of statisticians should be strengthened. Conversely, if the objective is to assess the practical value of acupuncture within an integrated rehabilitation program, add‐on designs remain acceptable, provided that the type, dose, frequency, and implementation procedures of concomitant treatments are prespecified and accurately documented. Otherwise, the introduction of confounding factors will weaken the interpretability of the findings. The SHARE framework may be used to guide study design and reporting, thereby improving transparency and reproducibility (Liu et al. 2025).

Finally, insufficient follow‐up also limits the clinical interpretation of existing findings. For key outcomes such as aspiration, pneumonia, safety, and long‐term functional recovery, future studies should perform a priori sample size calculations and establish appropriate follow‐up durations to improve the stability and clinical interpretability of the results (Dziewas et al. 2021; Hotz et al. 2025). Inadequate follow‐up not only hampers the assessment of the durability of treatment effects but also limits observation of real‐world functional outcomes. For patients with PSD, short‐term improvement in swallowing function does not necessarily translate into long‐term recovery of oral intake, reduced pneumonia risk, or improved quality of life; therefore, greater emphasis should be placed on medium‐ and long‐term follow‐up in future studies (Félix et al. 2025).

Taken together, due to limitations related to add‐on intervention designs, comparator settings, and follow‐up duration, current studies of acupuncture for PSD largely demonstrate only short‐term functional improvement and a favorable trend. However, evidence regarding patient‐important outcomes and long‐term clinical value remains insufficient. This is one of the key reasons why the current evidence is not yet adequate to support strong clinical recommendations regarding the long‐term effectiveness of acupuncture for PSD.

### 4.3 Core Acupoint Characteristics and Clinical Implications

Beyond outcome selection and study design, prescription pattern analysis offers additional information on the intervention's structure. As the primary units of analysis in this study were SRs/MAs, the evidence mapping component mainly reflects the quantity and quality of secondary evidence, outcome coverage, and overlap among reviews. However, SRs/MAs alone cannot fully characterize the specific composition of acupuncture interventions used in primary trials. The clinical reproducibility of acupuncture treatment depends not only on evidence quality but also on the stability of prescription components. Building on this, we further extracted acupuncture prescription information from full‐text primary RCTs included in eligible SRs/MAs and conducted pattern mining to characterize recurrent acupuncture intervention patterns within the current body of secondary evidence. Co‐occurrence network analysis revealed GB20, RN23, EX‐HN12, and EX‐HN13 as high‐frequency and closely connected core acupoints in the secondary evidence base for acupuncture treatment of PSD. This suggests a concentrated trend in acupoint selection in current PSD acupuncture research, with specific local and distal regulatory acupoints repeatedly combined across studies.

It should be noted that the core acupoints identified in this study were not derived directly from all primary RCTs retrieved in the initial database search. Instead, they came from primary trials already included in SRs/MAs, thus forming part of the secondary evidence base. Therefore, these acupoints reflect commonly reported prescription patterns within the existing secondary evidence. This finding is broadly consistent with the data‐mining results of Wu et al. (2024) based on 149 RCTs. Building on this evidence, another study further developed a standardized acupuncture regimen centered on Fengchi, Lianquan, Jinjin, and Yuye, and preliminarily explored its clinical effects and related mechanisms through an RCT and MRI study (Wu 2025).

Previous research suggests that acupuncture at RN23 and adjacent acupoints may improve swallowing‐related motor function by increasing the superior and anterior displacement of the hyoid bone and thyroid cartilage, thereby enhancing lower‐pharyngeal airway protection and facilitating upper esophageal sphincter opening (Shen et al. 2021). Liu and Qiu (2014) reported that stimulation of EX‐HN12 and EX‐HN13 may be associated with improved tongue mobility and enhanced bolus control. Using functional magnetic resonance imaging, Sun et al. (2025) observed that stimulation of Fengchi and tongue‐related acupoints could immediately modulate brain functional networks and enhance functional connectivity within the sensorimotor network. Taken together, the repeated use of GB20, RN23, EX‐HN12, and EX‐HN13 in related studies may reflect their potential combined roles in local regulation of swallowing movement and central modulation of functional brain networks.

Further integration of meridian attribution, stimulation‐site distribution, and specific acupoint attributes reveals a clear hierarchical combination pattern in current acupuncture prescriptions for PSD. The stimulation sites were primarily concentrated in the suprahyoid region, posterior neck, and distal upper limb. This indicates that existing studies emphasize both local intervention targeting swallowing execution and acupoint selection in head–neck regions, complemented by distal synergistic acupoints. Meridian attribution was concentrated primarily in the SJ, GB, ST, and RN meridians. Among these, RN was primarily associated with local acupoint selection in the anterior pharyngeal region; GB and SJ were frequently linked to the head, nape, and lateral neck regions; and ST included both head–face–neck acupoints and distal limb acupoints. Regarding specific acupoint attributes, CP, LCP, and ECP were commonly used. This pattern was consistent with the distributions of meridian attribution and stimulation sites, suggesting that current acupuncture prescriptions for PSD emphasize not only spatial compatibility between local and distal acupoints but also the synergistic use of different categories of specific acupoints.

Overall, acupuncture research on PSD has developed a prescription pattern integrating local throat region acupoints, head–neck‐related acupoints, and distal synergistic acupoints, broadly consistent with acupoint selection patterns summarized in previous reviews (Guo et al. 2024). Threshold sensitivity analysis showed that several core associations persisted across alternative parameter settings, although rule retention decreased under stricter confidence thresholds. Taken together, the existing secondary evidence suggests that acupuncture treatment for PSD has formed a prescription structure centered on core acupoints and aligned with meridian attribution and stimulation‐site distribution. The critical next step is to further validate these combination patterns under standardized outcome frameworks and rigorous study designs. Importantly, the frequency and association strength of these acupoints and combinations reflect patterns of use and co‐occurrence in the existing literature and should not be interpreted as evidence of superior clinical efficacy. The identified combinations should therefore be regarded as candidate prescription patterns for future standardization and prospective validation rather than as current evidence‐based treatment recommendations. Future studies should conduct multicenter, well‐designed confirmatory trials focusing on these core combinations and evaluate their clinical value using both subjective and objective outcomes.

### 4.4 Limitations and Future Perspectives

This study has several limitations. First, most included studies originated from China, which may limit the international generalizability of the findings; potential regional or language‐related bias therefore cannot be excluded. Second, prescription pattern mining relied on primary RCTs already included in eligible SRs/MAs, rather than on the complete set of RCTs retrieved directly from all databases. Consequently, the results should be interpreted as patterns frequently reported within the existing body of secondary evidence, rather than as standardized prescriptions representing all RCTs of acupuncture for PSD. Furthermore, since prescription data were extracted from only 470 de‐duplicated RCTs with accessible full texts (out of 779 unique RCTs), the identified high‐frequency combination patterns may have been influenced by literature availability. Third, we did not further assess the risk of bias or certainty of evidence for the primary RCTs used in prescription pattern mining. Consequently, these patterns primarily reflect high‐frequency prescription characteristics within the existing secondary evidence base and should not be directly regarded as standardized regimens supported by high‐quality evidence. Fourth, overall AMSTAR 2 confidence ratings were sensitive to the classification of Item 7. We therefore report both the original AMSTAR 2 critical‐domain configuration and an alternative configuration in which Item 7 was not treated as critical. The original configuration was retained as the reference assessment, whereas the alternative configuration was used to examine the influence of Item 7 classification on the overall ratings. Accordingly, comparisons with other studies should primarily refer to the original AMSTAR 2 configuration.

Fifth, further stratified analyses of specific clinical parameters, such as the type and dosage of drugs used in comparator groups and the details of co‐interventions, were not undertaken in this study, leaving some sources of heterogeneity unexplained. Future studies should aim for more complete access to the full texts of primary trials and perform stratified analyses based on key clinical parameters. Where appropriate, data‐driven analytical methods could also be incorporated to improve the efficiency of evidence synthesis, prescription pattern mining, and regimen optimization (Liu and Gong 2024). Relatedly, prescription‐pattern analyses were not stratified by acupuncture modality, intervention timing, or TCM syndrome differentiation because these characteristics were incompletely or inconsistently reported across the underlying trials. Therefore, the identified patterns should be interpreted as aggregate prescription characteristics rather than subgroup‐specific treatment patterns.

## 5 Conclusions

Current secondary evidence suggests that acupuncture, primarily as an adjunctive intervention, may improve swallowing‐related outcomes in PSD; however, the underlying evidence is of limited methodological quality and low certainty, and evidence regarding aspiration/pneumonia, nutritional status, safety, and long‐term outcomes remains insufficient. By integrating evidence mapping and prescription pattern mining, this study characterized the evidence structure of acupuncture for PSD and identified recurrent core prescription patterns within the existing body of secondary evidence. These findings may inform future study design, regimen selection, and standardized validation. Future research should prioritize the development of a COS for acupuncture studies in PSD and conduct rigorously designed international multicenter RCTs, using sham acupuncture where appropriate, with standardized outcome measures, objective instrumental assessments, and adequate long‐term follow‐up.

## Author Contributions

G.L. contributed to conceptualization, methodology, data extraction, formal analysis, visualization, and drafting of the manuscript. M.W. contributed to supervision, methodology, validation, and critical revision of the manuscript. J.Z. contributed to literature search, data extraction, methodology, and validation. Y.L. contributed to literature search and data extraction. Y.W. contributed to data extraction, statistical analysis, and visualization. Y.S. contributed to literature search and data extraction. Z.D. contributed to supervision, project administration, and critical revision of the manuscript. X.W. contributed to data extraction, statistical analysis, and validation. L.Z. contributed to supervision, project administration, funding acquisition, and critical revision of the manuscript. All authors reviewed and approved the final version of the manuscript.

## Funding

This work was supported by the Natural Science Foundation of Heilongjiang Province (Grant No. JQ2025H002). The funder had no role in the study design, data collection, data analysis, interpretation of the results, manuscript preparation, or decision to submit the article for publication.

## Disclosure

This overview was registered in PROSPERO on June 19, 2026 (CRD420261428106). No separately accessible full protocol was prepared. This overview was reported in accordance with the PRISMA 2020 statement. The completed PRISMA checklist is provided as Supporting Information.

## Ethics Statement

Ethics approval was not required for this overview because it was based exclusively on previously published systematic reviews and meta‐analyses and did not involve new human participants, animal experiments, or individual patient data.

## Conflicts of Interest

The authors declare no conflicts of interest.

## Supporting information



Supplementary Appendix: Search strategies, characteristics of included reviews, AMSTAR 2 assessment, GRADE assessment, corrected covered area calculation, evidence mapping details, and acupoint prescription pattern mining results.
Supplementary Code S1: R code for association rule mining, threshold sensitivity analysis, and graphical visualization.
PRISMA 2020 Checklist: Completed PRISMA 2020 checklist for this overview.

**Supplementart information**: brb371887‐sup‐0002‐S1.R

## Data Availability

All data generated or analyzed during this study are included in this article and its Supporting Information.
