# Supplementary material for: Evidence Mapping, Certainty Appraisal, and Acupoint Prescription Pattern Mining of Acupuncture for Poststroke Dysphagia: An Overview of Systematic Reviews
Source: Brain Behav. 2026 Nov 1;16(11):e71887. doi: 10.1002/brb3.71887 (PMC13724206; doi:10.1002/brb3.71887)
Supplement: Supplementary file 1 [file BRB3-16-e71887-s002.docx]

Supplementary appendix

**Evidence Mapping, Certainty Appraisal, and Acupoint Prescription Pattern Mining of Acupuncture for Post-Stroke Dysphagia: An Overview of Systematic Reviews**

This supplementary appendix includes Supplementary Tables S1–S9 and Supplementary Figures S1–S4.

****Table S1.**** Full search strategies and database-specific retrieval results

Search Date: All databases were searched from inception to 12 June 2026.

| **Database** | **Search term** | **Results** |
| --- | --- | --- |
| PubMed | #1:(((((((((((((((((((((((((((((((((((((Stroke,Acute[Title/Abstract])OR (Brain Infarction[Title/Abstract]))OR(Cerebral Infarct[Title/Abstract]))OR(Intracerebral Hemorrhage[Title/Abstract]))OR(Cerebral Hemorrhage[Title/Abstract]))OR(Cerebral Brain Hemorrhage[Title/Abstract]))OR(Hemorrhagic Stroke[Title/Abstract]))OR(Hypertensive Intracerebral Hemorrhage[Title/Abstract]))OR(Hemorrhage, Cerebrum[Title/Abstract]))OR(Cerebrum Hemorrhage[Title/Abstract]))OR(Cerebral Parenchymal Hemorrhage[Title/Abstract]))OR(Hemorrhage, Cerebral Parenchymal[Title/Abstract]))OR(Parenchymal Hemorrhage, Cerebral[Title/Abstract]))OR(Hemorrhage, Intracerebral[Title/Abstract]))OR(Hemorrhage, Cerebral[Title/Abstract]))OR(Cerebral Hemorrhage[Title/Abstract])) OR(Brain Hemorrhage, Cerebral[Title/Abstract]))OR(Hemorrhage, Cerebral Brain[Title/Abstract]))OR(Cerebrovascular Accident[Title/Abstract]))OR(Acute Cerebrovascular Accident[Title/Abstract]))OR(CVA[Title/Abstract]))OR(Vascular Accident, Brain[Title/Abstract]))OR(Brain Vascular Accident[Title/Abstract]))OR(Stroke, Cerebral[Title/Abstract]))OR (Stroke, Cerebrovascular[Title/Abstract]))OR(Brain Ischemia[Title/Abstract]))OR(Thrombotic Stroke[Title/Abstract]))OR (Ischemic Stroke[Title/Abstract]))OR(Wake up Stroke[Title/Abstract]))OR(Embolic Stroke[Title/Abstract]))OR (Acute Stroke[Title/Abstract]))OR(Cerebral Stroke[Title/Abstract])) OR(Cerebrovascular Stroke[Title/Abstract]))OR(Apoplexy, Cerebrovascular[Title/Abstract]))OR(Cerebrovascular Apoplexy[Title/Abstract]))OR(Apoplexy[Title/Abstract]))OR (Stroke[Title/Abstract]))OR(Stroke[MeSH Terms])  #2: (((((((((Deglutition Disorders[MeSH Terms]) OR (Deglutition Disorder[Title/Abstract])) OR (Disorders, Deglutition[Title/Abstract])) OR (Dysphagia[Title/Abstract])) OR (Swallowing Disorders[Title/Abstract])) OR (Swallowing Disorder[Title/Abstract])) OR (Oropharyngeal Dysphagia[Title/Abstract])) OR (Dysphagia, Oropharyngeal[Title/Abstract])) OR (Esophageal Dysphagia[Title/Abstract])) OR (Dysphagia, Esophageal[Title/Abstract])  #3:((Acupuncture[MeSH Terms])OR(Moxibustion[MeSH Terms])) OR(Acupuncture Therapy[MeSH Terms])  #4: "Meta-Analysis" [Publication Type] OR Meta-Analysis [Title/Abstract] OR "Systematic Review" [Publication Type] OR Systematic Review [Title/Abstract]  #5:((((((((((((((((((((((((((((((((((((((((Stroke,Acute[Title/Abstract])OR (Brain Infarction[Title/Abstract]))OR(Cerebral Infarct[Title/Abstract]))OR(Intracerebral Hemorrhage[Title/Abstract]))OR(Cerebral Hemorrhage[Title/Abstract]))OR(Cerebral Brain Hemorrhage[Title/Abstract]))OR(Hemorrhagic Stroke[Title/Abstract]))OR(Hypertensive Intracerebral Hemorrhage[Title/Abstract]))OR(Hemorrhage, Cerebrum[Title/Abstract]))OR(Cerebrum Hemorrhage[Title/Abstract]))OR(Cerebral Parenchymal Hemorrhage[Title/Abstract]))OR(Hemorrhage, Cerebral Parenchymal[Title/Abstract]))OR(Parenchymal Hemorrhage, Cerebral[Title/Abstract]))OR(Hemorrhage, Intracerebral[Title/Abstract]))OR(Hemorrhage, Cerebral[Title/Abstract]))OR(Cerebral Hemorrhage[Title/Abstract])) OR(Brain Hemorrhage, Cerebral[Title/Abstract]))OR(Hemorrhage, Cerebral Brain[Title/Abstract]))OR(Cerebrovascular Accident[Title/Abstract]))OR(Acute Cerebrovascular Accident[Title/Abstract]))OR(CVA[Title/Abstract]))OR(Vascular Accident, Brain[Title/Abstract]))OR(Brain Vascular Accident[Title/Abstract]))OR(Stroke, Cerebral[Title/Abstract]))OR (Stroke, Cerebrovascular[Title/Abstract]))OR(Brain Ischemia[Title/Abstract]))OR(Thrombotic Stroke[Title/Abstract]))OR (Ischemic Stroke[Title/Abstract]))OR(Wake up Stroke[Title/Abstract]))OR(Embolic Stroke[Title/Abstract]))OR (Acute Stroke[Title/Abstract]))OR(Cerebral Stroke[Title/Abstract])) OR(Cerebrovascular Stroke[Title/Abstract]))OR(Apoplexy, Cerebrovascular[Title/Abstract]))OR(Cerebrovascular Apoplexy[Title/Abstract]))OR(Apoplexy[Title/Abstract]))OR (Stroke[Title/Abstract]))OR(Stroke[MeSH Terms])) AND ((((((((((Deglutition Disorders[MeSH Terms]) OR (Deglutition Disorder[Title/Abstract])) OR (Disorders, Deglutition[Title/Abstract])) OR (Dysphagia[Title/Abstract])) OR (Swallowing Disorders[Title/Abstract])) OR (Swallowing Disorder[Title/Abstract])) OR (Oropharyngeal Dysphagia[Title/Abstract])) OR (Dysphagia, Oropharyngeal[Title/Abstract])) OR (Esophageal Dysphagia[Title/Abstract])) OR (Dysphagia, Esophageal[Title/Abstract]))) AND (((Acupuncture[MeSH Terms])OR(Moxibustion[MeSH Terms])) OR(Acupuncture Therapy[MeSH Terms]))) AND ("Meta-Analysis" [Publication Type] OR Meta-Analysis [Title/Abstract] OR "Systematic Review" [Publication Type] OR Systematic Review [Title/Abstract]) | 37 |
| Web of Science | (("Stroke" OR "Acute Stroke" OR "Ischemic Stroke" OR "Hemorrhagic Stroke" OR "Cerebral Stroke" OR "Cerebrovascular Stroke" OR "Cerebrovascular Accident" OR "Acute Cerebrovascular Accident" OR "Apoplexy" OR "Cerebrovascular Apoplexy" OR "Vascular Accident" OR "CVA" OR "Brain Vascular Accident" OR "Post-stroke")  AND  ("Deglutition Disorders" OR "Deglutition Disorder" OR "Disorders, Deglutition" OR "Dysphagia" OR "Swallowing Disorders" OR "Swallowing Disorder" OR "Oropharyngeal Dysphagia" OR "Dysphagia, Oropharyngeal" OR "Esophageal Dysphagia" OR "Dysphagia, Esophageal")  AND  ("Acupuncture" OR "Acupuncture Therapy" OR "Acupuncture Treatment" OR "Treatment, Acupuncture" OR "Therapy, Acupuncture" OR "Manual Acupuncture" OR "Acupuncture-moxibustion" OR "Moxibustion" OR "Moxabustion" OR "Warm needle" OR "Warm Acupuncture" OR "Thermoacupuncture" OR "Electroacupuncture" OR "Electro-acupuncture" OR "electric acupuncture" OR "Acupuncture Point" OR "Point, Acupuncture" OR "Acupoint" OR "Pharmacopuncture" OR "Pharmacoacupuncture Treatment" OR "Treatment, Pharmacoacupuncture" OR "Pharmacoacupuncture Therapy" OR "Therapy, Pharmacoacupuncture" OR "Acupoint injection" OR "acupuncture injection" OR "pharmaco-acupuncture" OR "auricular needle" OR "Acupunctures, Ear" OR "Ear Acupuncture" OR "Ear needle" OR "earlobe acupuncture" OR "Auricular Acupuncture" OR "Auricular therapy" OR "Acupuncture, Auricular" OR "auricular plaster therapy" OR "auricular point sticking" OR "Auricular pressure" OR "Fire needle" OR "Fire acupuncture" OR "Acupoint catgut embedding" OR "Scalp acupuncture" OR "Scalp needle" OR "Scalp electroacupuncture" OR "scalp stimulation" OR "Eye needle" OR "Eye acupuncture" OR "Abdominal acupuncture" OR "Abdominal needle" OR "filiform needle" OR "silver needle" OR "three-edged needle" OR "intradermal needle" OR "Point application" OR "needle-embedding" OR "Catgut Embedding" OR "pricking therapy" OR "point injection" OR "Skin Acupuncture" OR "transcutaneous electrical acupoint stimulation" OR "electrical acupoint stimulation")  AND  ("Meta-Analysis" OR "Systematic Review")) | 33 |
| Embase | #1'acupuncture':ti,ab,kw OR 'acupuncture therapy':ti,ab,kw OR 'acupuncture treatment':ti,ab,kw OR 'treatment,acupuncture':ti,ab,kw OR 'therapy,acupuncture':ti,ab,kw OR 'manual acupuncture':ti,ab,kw OR 'acupuncture-moxibustion':ti,ab,kw OR 'moxibustion':ti,ab,kw OR 'moxabustion':ti,ab,kw OR 'warm needle':ti,ab,kw OR 'warm acupuncture':ti,ab,kw OR 'thermoacupuncture':ti,ab,kw OR 'electroacupuncture':ti,ab,kw OR 'electro-acupuncture':ti,ab,kw OR 'electric acupuncture':ti,ab,kw OR 'point,acupuncture':ti,ab,kw OR 'pharmacopuncture':ti,ab,kw OR 'pharmacoacupuncture treatment':ti,ab,kw OR 'treatment, pharmacoacupuncture':ti,ab,kw OR 'pharmacoacupuncture therapy':ti,ab,kw OR 'therapy, pharmacoacupuncture':ti,ab,kw OR 'acupoint injection':ti,ab,kw OR 'acupuncture injection':ti,ab,kw OR 'pharmaco-acupuncture':ti,ab,kw OR 'auricular needle':ti,ab,kw OR 'acupunctures, ear':ti,ab,kw OR 'ear acupuncture':ti,ab,kw OR 'ear needle':ti,ab,kw OR 'earlobe acupuncture':ti,ab,kw OR 'auricular acupuncture':ti,ab,kw OR 'auricular therapy':ti,ab,kw OR 'acupuncture, auricular':ti,ab,kw OR 'auricular plaster therapy':ti,ab,kw OR 'auricular point sticking':ti,ab,kw OR 'auricular pressure':ti,ab,kw OR 'fire needle':ti,ab,kw OR 'fire acupuncture':ti,ab,kw OR 'acupoint catgut embedding':ti,ab,kw OR 'scalp acupuncture':ti,ab,kw OR 'scalp needle':ti,ab,kw OR 'scalp electroacupuncture':ti,ab,kw OR 'scalp stimulation':ti,ab,kw OR 'eye needle':ti,ab,kw OR 'eye acupuncture':ti,ab,kw OR 'abdominal acupuncture':ti,ab,kw OR 'abdominal needle':ti,ab,kw OR 'filiform needle':ti,ab,kw OR 'silver needle':ti,ab,kw OR 'three-edged needle':ti,ab,kw OR 'intradermal needle':ti,ab,kw OR 'point application':ti,ab,kw OR 'needle-embedding':ti,ab,kw OR 'catgut embedding':ti,ab,kw OR 'pricking therapy':ti,ab,kw OR 'point injection':ti,ab,kw OR 'skin acupuncture':ti,ab,kw OR 'transcutaneous electrical acupoint stimulation':ti,ab,kw OR 'electrical acupoint stimulation':ti,ab,kw OR 'acupuncture point':ti,ab,kw OR 'acupoint':ti,ab,kw  #2'stroke':ti,ab,kw OR 'acute stroke':ti,ab,kw OR 'ischemic stroke':ti,ab,kw OR 'hemorrhagic stroke':ti,ab,kw OR 'cerebral stroke':ti,ab,kw OR 'cerebrovascular stroke':ti,ab,kw OR 'cerebrovascular accident':ti,ab,kw OR 'acute cerebrovascular accident':ti,ab,kw OR 'apoplexy':ti,ab,kw OR 'cerebrovascular apoplexy':ti,ab,kw OR 'vascular accident':ti,ab,kw OR 'cva':ti,ab,kw OR 'brain vascular accident':ti,ab,kw OR 'post-stroke':ti,ab,kw  #3'deglutition disorders':ti,ab,kw OR 'deglutition disorder':ti,ab,kw OR 'disorders, deglutition':ti,ab,kw OR 'dysphagia':ti,ab,kw OR 'swallowing disorders':ti,ab,kw OR 'swallowing disorder':ti,ab,kw OR 'oropharyngeal dysphagia':ti,ab,kw OR 'dysphagia, oropharyngeal':ti,ab,kw OR 'esophageal dysphagia':ti,ab,kw OR 'dysphagia, esophageal':ti,ab,kw  #4: 'Meta-Analysis':ti,ab,kw OR 'Systematic Review':ti,ab,kw  #5: #1 AND #2 AND #3 AND #4 | 37 |
| Cochrane Library | #1:(Acupuncture OR Acupuncture Therapy OR Acupuncture Treatment OR Treatment, Acupuncture OR Therapy, Acupuncture OR Manual Acupuncture OR Acupuncture-moxibustion OR Moxibustion OR Moxabustion OR Warm needle OR Warm Acupuncture OR Thermoacupuncture OR Electroacupuncture OR Electro-acupuncture OR electric acupuncture OR Acupuncture Point OR Point, Acupuncture OR Acupoint OR Pharmacopuncture OR Pharmacoacupuncture Treatment OR Treatment, Pharmacoacupuncture OR Pharmacoacupuncture Therapy OR Therapy, Pharmacoacupuncture OR Acupoint injection OR acupuncture injection OR pharmaco-acupuncture OR auricular needle OR Acupunctures, Ear OR Ear Acupuncture OR Ear needle OR earlobe acupuncture OR Auricular Acupuncture OR Auricular therapy OR Acupuncture, Auricular OR auricular plaster therapy OR auricular point sticking OR Auricular pressure OR Fire needle OR Fire acupuncture OR Acupoint catgut embedding OR Scalp acupuncture OR Scalp needle OR Scalp electroacupuncture OR scalp stimulation OR Eye needle OR Eye acupuncture OR Abdominal acupuncture OR Abdominal needle OR filiform needle OR silver needle OR three-edged needle OR intradermal needle OR Point application OR needle-embedding OR Catgut Embedding OR pricking therapy OR point injection OR Skin Acupuncture OR transcutaneous electrical acupoint stimulation OR electrical acupoint stimulation OR Acupuncture Point OR Acupoint):ti,ab,kw  #2:(“Stroke” OR “Acute Stroke” OR “Ischemic Stroke” OR “Hemorrhagic Stroke” OR “Cerebral Stroke ” OR “Cerebrovascular Stroke” OR “Cerebrovascular Accident” OR “Acute Cerebrovascular Accident ” OR “Apoplexy” OR “Cerebrovascular Apoplexy” OR “Vascular Accident” OR “CVA” OR “Brain Vascular Accident” OR “Post-stroke”):ti,ab,kw  #3:(“Deglutition Disorders” OR “Deglutition Disorder” OR “Disorders, Deglutition” OR “Dysphagia” OR “Swallowing Disorders” OR “Swallowing Disorder” OR “Oropharyngeal Dysphagia” OR “Dysphagia, Oropharyngeal” OR “Esophageal Dysphagia” OR “Dysphagia, Esophageal”):ti,ab,kw  #4: (“Meta-Analysis” OR “Systematic Review”):ti,ab,kw  #5: #1 AND #2 AND #3 AND #4 | 0 |
| CNKI | (SU='吞咽困难' +'吞咽障碍' AND TKA='吞咽困难' +'吞咽障碍')AND(SU='中风'+'卒中'+'脑梗死'+'脑梗塞'+'脑出血' AND TKA='中风'+'卒中'+'脑梗死'+'脑梗塞'+'脑出血')AND (SU='针刺'+'电针'+'针灸'+'刺法'+'经皮穴位电刺激'+'隔姜灸'+'隔附子饼灸'+'体针'+'耳针'+'头针'+'毫针隔盐灸'+'耳穴贴压'+'热敏灸'+'梅花针'+'针刺治疗'+'针灸疗法'+'穴位埋线'+'火针'+'穴位注射'+'艾灸'+'灸法'+'灸疗'+'灸术'+'温针'+'针法'+'三棱针'+'皮肤针'+'芒针'+'眼针'+'手针'+'足针'+'腕踝针'+'平衡针'+'撳针'+'皮内针'+'腹针'+'舌针'+'项针'AND TKA='针刺'+'电针'+'针灸'+'刺法'+'经皮穴位电刺激'+'隔姜灸'+'隔附子饼灸'+'体针'+'耳针'+'头针'+'毫针隔盐灸'+'耳穴贴压'+'热敏灸'+'梅花针'+'针刺治疗'+'针灸疗法'+'穴位埋线'+'火针'+'穴位注射'+'艾灸'+'灸法'+'灸疗'+'灸术'+'温针'+'针法'+'三棱针'+'皮肤针'+'芒针'+'眼针'+'手针'+'足针'+'腕踝针'+'平衡针'+'针'+'皮内针'+'腹针'+'舌针'+'项针') (SU='meta分析' +'系统评价' AND TKA='meta分析' +'系统评价') | 60 |
| VIP | M=(针刺 OR 电针 OR 针灸 OR 刺法 OR 经皮穴位电刺激 OR 隔姜灸 OR 隔附子饼灸 OR 体针 OR 耳针 OR 头针 OR 毫针隔盐灸 OR 耳穴贴压 OR 热敏灸 OR 梅花针 OR 针刺治疗 OR 针灸疗法 OR 穴位埋线 OR 火针 OR 穴位注射 OR 艾灸 OR 灸法 OR 灸疗 OR 灸术 OR 温针 OR 针法 OR 三棱针 OR 皮肤针 OR 芒针 OR 眼针 OR 手针 OR 足针 OR 腕踝针 OR 平衡针 OR 揿针 OR 皮内针 OR 腹针 OR 舌针 OR 项针) AND M=(中风 OR 卒中 OR 脑梗死 OR 脑梗塞 OR 脑出血) AND M=(吞咽困难 OR 吞咽障碍) AND M=(meta分析 OR 系统评价) | 34 |
| CBM | ("针刺"[核心字段:智能] OR "电针"[核心字段:智能] OR "针灸"[核心字段:智能] OR "刺法"[核心字段:智能] OR "经皮穴位电刺激"[核心字段:智能] OR "隔姜灸"[核心字段:智能] OR "隔附子饼灸"[核心字段:智能] OR "体针"[核心字段:智能] OR "耳针"[核心字段:智能] OR "头针"[核心字段:智能] OR "毫针隔盐灸"[核心字段:智能] OR "耳穴贴压"[核心字段:智能] OR "热敏灸"[核心字段:智能] OR "梅花针"[核心字段:智能] OR "针刺治疗"[核心字段:智能] OR "针灸疗法"[核心字段:智能] OR "穴位埋线"[核心字段:智能] OR "火针"[核心字段:智能] OR "穴位注射"[核心字段:智能] OR "艾灸"[核心字段:智能] OR "灸法"[核心字段:智能] OR "灸疗"[核心字段:智能] OR "灸术"[核心字段:智能] OR "温针"[核心字段:智能] OR "针法"[核心字段:智能] OR "三棱针"[核心字段:智能] OR "皮肤针"[核心字段:智能] OR "芒针"[核心字段:智能] OR "眼针"[核心字段:智能] OR "手针"[核心字段:智能] OR "足针"[核心字段:智能] OR "腕踝针"[核心字段:智能] OR "平衡针"[核心字段:智能] OR "揿针"[核心字段:智能] OR "皮内针"[核心字段:智能] OR "腹针"[核心字段:智能] OR "舌针"[核心字段:智能] OR "项针"[核心字段:智能] ) AND ("中风"[核心字段:智能] OR "卒中"[核心字段:智能] OR "脑梗死"[核心字段:智能] OR "脑梗塞"[核心字段:智能] OR "脑出血"[核心字段:智能]) AND ("吞咽困难"[核心字段:智能] OR "吞咽障碍"[核心字段:智能]) AND ("meta分析"[核心字段:智能] OR "系统评价"[核心字段:智能]) | 45 |
| 万方 | 主题:("针刺" OR "电针" OR "针灸" OR "刺法" OR "经皮穴位电刺激" OR "隔姜灸" OR "隔附子饼灸" OR "体针" OR "耳针" OR "头针" OR "毫针隔盐灸" OR "耳穴贴压" OR "热敏灸" OR "梅花针" OR "针刺治疗" OR "针灸疗法" OR "穴位埋线" OR "火针" OR "穴位注射" OR "艾灸" OR "灸法" OR "灸疗" OR "灸术" OR "温针" OR "针法" OR "三棱针" OR "皮肤针" OR "芒针" OR "眼针" OR "手针" OR "足针" OR "腕踝针" OR "平衡针" OR "揿针" OR "皮内针" OR "腹针" OR "舌针" OR "项针" ) AND 主题:("中风" OR "卒中" OR "脑梗死" OR "脑梗塞" OR "脑出血") AND 主题:("吞咽困难" OR "吞咽障碍" )AND 主题:("meta分析" OR "系统评价" ) | 64 |
| OpenGrey | #1 Stroke-related terms:  stroke OR "post-stroke" OR "post stroke" OR "cerebrovascular accident" OR "brain infarction" OR "cerebral infarction" OR "ischemic stroke" OR "haemorrhagic stroke" OR "hemorrhagic stroke" OR "intracerebral hemorrhage" OR "cerebral hemorrhage" OR apoplexy  #2 Dysphagia-related terms:  dysphagia OR "deglutition disorder" OR "deglutition disorders" OR "swallowing disorder" OR "swallowing disorders" OR "oropharyngeal dysphagia" OR "esophageal dysphagia"  #3 Acupuncture-related terms:  acupuncture OR electroacupuncture OR "electro-acupuncture" OR "scalp acupuncture" OR "tongue acupuncture" OR "eye acupuncture" OR "acupuncture therapy" OR moxibustion OR "warm needle" OR "warm-needle acupuncture"  #4 Systematic review/meta-analysis terms:  "systematic review" OR "meta-analysis" OR "meta analysis" OR review  #5 Final search:  #1 AND #2 AND #3 AND #4 | 0 |
| Total |  | 310 |

****Table S2.**** Cross-language matching and final unique RCT correspondence

| **trial** | **year** | **author** | **records CN** | **records EN** | **labels** |
| --- | --- | --- | --- | --- | --- |
| 13 | 2014 | Tong S | 3 | 1 | Tong S (2014) - Clinical observation of acupuncture therapy and swallowing functional rehabilitation for dysphagia a |
| 53 | 2009 | LV H | 3 | 1 | LV H (2009) - Randomized controlled trials of electroacupuncture for the dysphagia in stroke patients |
| 132 | 2008 | Cao Q | 2 | 1 | Cao Q (2008) - Treatment of 60 cases of dysphagia after stroke by head electroacupuncture combined with swallowing |
| 240 | 2017 | Zhen WJ | 3 | 1 | Zhen WJ (2017) - Observation on Therapeutic Effect of Acupuncture Synchronous Swallowing Rehabilitation Training on S |
| 332 | 2016 | Chen XM | 4 | 1 | Chen XM (2016) - Xingnaokaiqiao acupuncture therapy for dysphagia after stroke in 59 cases |
| 384 | 2016 | CHEN L; Chen L | 1 | 1 | Chen L (2016) - Additional effects of acupuncture on early comprehensive rehabilitation in patients with mild to mod |
| 421 | 2020 | Gao JX | 1 | 3 | Gao JX (2020) - Therapeutic effect of nape cluster acupuncture combined with swallowing function training on post-st |
| 434 | 2004 | Han JC | 1 | 1 | Han JC (2004) - Observation of the effect of acupuncture on deglutition difficulty in bulbar palsy patients |
| 462 | 2011 | Jin DQ | 1 | 1 | Jin DQ (2011) - Clinical study on acupuncturing lianquan combined with qingyin liyan powder to treat dysphagia after |
| 497 | 2012 | DANDAN LIU; Liu DD | 1 | 2 | Liu DD (2012) - Influence of acupuncture on remodeling of swallowing functions for patients with pseudobulbar palsy |
| 559 | 2007 | WANG FENGYAN; Wang FY | 1 | 1 | Wang FY (2007) - Clinical research on treatment of dysphagia with neck acupuncture |
| 594 | 2019 | Wu W-b; Wu WB | 1 | 2 | Wu W-b (2019) - Relieving throat and opening orifice acupuncture therapy for the post-stroke dysphagia \| Wu WB (2019) - Relieving throat and opening orifice acupuncture therapy for the post-stroke dysphagia |
| 602 | 2016 | Xia W | 1 | 2 | Xia W (2016) - Does the addition of specific acupuncture to standard swallowing training improve outcomes in patien |
| 604 | 2011 | Xie Y | 1 | 2 | Xie Y (2011) - Effect of acupuncture on dysphagia of convalescent stroke patients |
| 625 | 2013 | Yin L-L; Yin LL | 8 | 3 | Yin L-L (2013) - Clinical study on acupuncture combined with rehabilitation therapy for 57 cases of swallowing disord \| Yin LL (2013) - A Clinical Study on Acupuncture Combined with Rehabilitation Therapy for 57 Cases of Swallowing Diso \| Yin LL (2013) - Clinical study on acupuncture combined with rehabilitation therapy for 57 cases of dysphagia after s |

****Table S3.**** Full-text articles excluded after eligibility assessment, with reasons

| **Reason for Exclusion** | **n** | **Excluded Studies** |
| --- | --- | --- |
| Not acupuncture | 24 | 1.Han CY, Zhao C, Wang XL, et al. Network meta-analysis of 13 intervention programs for post-stroke dysphagia [Chinese]. *Hu Li Yan Jiu*. 2020;34(11):1869-77.  2.Wang Y, Wang F, Wang W, et al. Meta-analysis of the efficacy of auricular therapy in patients with post-stroke dysphagia [Chinese]. *Dang Dai Hu Shi*. 2022;29(12):76-80.  3.Han AH, Cao Y, Wang QS, et al. Efficacy of hyperbaric oxygen therapy for post-stroke dysphagia: a meta-analysis [Chinese]. *Zhong Guo Gong Gong Wei Sheng*. 2018;34(9):1299-302.  4.Chen Q, Yun J, Chen YX, et al. Efficacy of transcutaneous electrical acupoint stimulation for post-stroke dysphagia: a meta-analysis [Chinese]. *Zhong Yi Lin Chuang Yan Jiu*. 2023;15(1):116-20.  5.Li CB, Yuan J, Liao CX, et al. Meta-analysis of efficacy and safety of moxibustion for post-stroke dysphagia [Chinese]. *Zhong Guo Zhong Yi Yao Tu Shu Qing Bao Za Zhi*. 2024;48(5):157-64.  6.Liu S, Pei ZW, Meng XM. Systematic review and meta-analysis of neuromuscular electrical stimulation for post-stroke dysphagia [Chinese]. *Zhong Guo Kang Fu*. 2019;34(8):426-31.  7.Wang JL, Dai XJ, Zhai XP, et al. Meta-analysis of clinical efficacy of neuromuscular electrical stimulation for post-stroke dysphagia [Chinese]. *Xun Zheng Hu Li*. 2018;4(1):9-13.  8.Wang JL, Dai XJ, Shi Q, et al. Meta-analysis of the effect of acupoint massage on post-stroke dysphagia [Chinese]. *Hu Li Yan Jiu*. 2020;34(5):821-7.  9.He J, Zheng M, He CQ, et al. Systematic review of acupoint stimulation therapy for post-stroke dysphagia [Chinese]. *Zhongguo Zhen Jiu*. 2009;29(1):66-71.  10.Xiao HF, Wang RR, Wang DQ. Meta-analysis of pharyngeal electrical stimulation for post-stroke dysphagia [Chinese]. *Zhong Guo Kang Fu Li Lun Yu Shi Jian*. 2020;26(11):1280-6.  11.Guo GZ, Han RS, Wang YQ, et al. Meta-analysis of repetitive transcranial magnetic stimulation for post-stroke dysphagia [Chinese]. *Wu Han Da Xue Xue Bao (Yi Xue Ban)*. 2018;39(5):855-60.  12.Yu MH, Li JW, Ye ZX, et al. Meta-analysis of the efficacy of repetitive transcranial magnetic stimulation for post-stroke dysphagia [Chinese]. *Shi Yong Lin Chuang Yi Yao Za Zhi*. 2021;16(6):311-5.  13.Liu L, Liu HB, Wang XL, et al. Systematic review of repetitive transcranial magnetic stimulation for post-stroke dysphagia [Chinese]. *Lin Chuang Shen Jing Bing Xue Za Zhi*. 2014;11(5):250-5.  14.Chu YQ, Zhou KY, Wang TY, et al. Meta-analysis of auricular point pressing therapy for post-stroke dysphagia [Chinese]. *Zhong Guo Zu Zhong Za Zhi*. 2022;17(12):1327-34.  15.Si H, Liao R, Yu Y, et al. A meta-analysis of acupoint application in the treatment of dysphagia after stroke. *Chin J Integr Nurs*. 2023;9(7):13-22.  16.Guo J, Chen C, Xie F. A meta-analysis of the effect of noninvasive brain stimulation on dysphagia in patients with cerebral stroke. *Altern Ther Health Med*. 2024. [Epub ahead of print].  17.Ebihara S, Naito T. A systematic review of reported methods of stimulating swallowing and of assessing swallowing-related outcomes. *Tohoku J Exp Med*. 2022;256(1):1-17. doi:10.1620/tjem.256.1  18.Wang L, Qiao J, Sun F, et al. Demographic and clinical factors associated with recovery of poststroke dysphagia: a meta-analysis. *Brain Behav*. 2023;13(6):e3033. doi:10.1002/brb3.3033  19.Wang Y, Xu L, Wang L, et al. Effects of transcutaneous neuromuscular electrical stimulation on post-stroke dysphagia: a systematic review and meta-analysis. *Front Neurol*. 2023;14:1163045. doi:10.3389/fneur.2023.1163045  20.Liu Y, Wang Y, Zhao X, et al. Efficacy of motor imagery in the treatment of poststroke dysphagia: a systematic review and meta-analysis. *Brain Behav*. 2025;15(9):e70826.  21.Wen X, Fan B, Zhan J, et al. Integrated analysis of the prevalence and influencing factors of poststroke dysphagia. *Eur J Med Res*. 2025;30(1):27-55. doi:10.1186/s40001-024-02220-x  22.Balcerak P, Corbiere S, Zubal R, et al. Post-stroke dysphagia: prognosis and treatment-a systematic review of RCT on interventional treatments for dysphagia following subacute stroke. *Front Neurol*. 2022;13:823189. doi:10.3389/fneur.2022.823189  23.Bath PM, Lee HS, Everton LF. Swallowing therapy for dysphagia in acute and subacute stroke. *Cochrane Database Syst Rev*. 2018;10:CD000323. doi:10.1002/14651858.CD000323.pub3  24.Qiao J, Ye QP, Wu ZM, et al. The effect and optimal parameters of repetitive transcranial magnetic stimulation on poststroke dysphagia: a meta-analysis of randomized controlled trials. *Front Neurosci*. 2022;16:845737. doi:10.3389/fnins.2022.845737 |
| Needling method not clearly defined or mixed with non-penetrating methods | 12 | 1.Meng D, Shang YB, Fu YF, et al. Clinical literature study of acupuncture and moxibustion for post-stroke dysphagia based on meta-analysis [Chinese]. *Zhong Guo Zhong Yi Yao Xian Dai Yuan Cheng Jiao Yu*. 2016;14(16):148-52.  2.Li JP. Systematic review of randomized controlled trials of Jin's Three-needle therapy for stroke [Chinese] [Dissertation]. Guangzhou: Guangzhou University of Chinese Medicine; 2016.  3.Fu YY, Dong LC, Pan JX, et al. Meta-analysis of tongue acupuncture and its combined therapy for post-stroke dysphagia [Chinese]. In: *Proc 2018 Shanghai Postgrad Acad Forum "Res Ideas Methods Integr Tradit Chin West Med"*. 2018:265-74.  4.Liang HH, Zhang P, Dai W, et al. "Tongguan Liqiao" acupuncture for post-stroke dysphagia: a systematic review and meta-analysis [Chinese]. In: *Proc 2024 China Tianjin Acad Conf*. 2024:1519-25. doi:10.26914/c.cnkihy.2024.039502  5.Wang H, Zhou HF, Li CR, et al. Meta-analysis of acupuncture combined with rehabilitation training for post-stroke dysphagia [Chinese]. *Zhen Jiu Lin Chuang Za Zhi*. 2019;35(10):75-81.  6.Sun TT. Meta-analysis of acupuncture combined with rehabilitation training for post-stroke dysphagia [Chinese] [Master's thesis]. Hefei: Anhui University of Chinese Medicine; 2025.  7.Wang YP. Systematic review of clinical efficacy of acupuncture combined with rehabilitation training for post-stroke dysphagia [Chinese] [Master's thesis]. Taiyuan: Shanxi University of Chinese Medicine; 2020.  8.Tian Y, Wang J, Song ZB, et al. Meta-analysis of acupuncture combined with swallowing training for post-stroke dysphagia [Chinese]. In: *Proc 4th TCM Sci Technol Achiev Forum*. 2012:244-9.  9.Bi SR. Meta-analysis of randomized controlled trials of acupuncture for post-stroke dysphagia [Chinese] [Master's thesis]. Guangzhou: Guangzhou University of Chinese Medicine; 2020.  10.Chen YY. Meta-analysis of acupuncture for post-stroke dysphagia [Chinese] [Master's thesis]. Changchun: Changchun University of Chinese Medicine; 2019.  11.Feng Y. Systematic review of syndrome-treatment rules and efficacy of post-stroke dysphagia [Chinese] [Master's thesis]. Jinan: Shandong University of Chinese Medicine; 2020.  12.Li H, Li J, Wang X, et al. A systematic review and meta-analysis of acupuncture in aspiration caused by post-stroke dysphagia. *Front Neurol*. 2024;15:1305056. doi:10.3389/fneur.2024.1305056 |
| Not SR/MA | 8 | 1.Yao LP, Zhang JY, Xi N, et al. Visual analysis of acupuncture for post-stroke dysphagia based on CiteSpace [Chinese]. *Zhong Yi Lin Chuang Yan Jiu*. 2024;16(9):16-22.  2.Shi L. Evidence-based medical study of acupuncture combined with rehabilitation training for post-stroke dysphagia [Chinese]. *Zhen Jiu Lin Chuang Za Zhi*. 2018;34(7):62-6.  3. Cordani C, Battel I, Del Furia MJ, Lazzarini SG, Negrini S, Arienti C. Dysphagia, dysphonia and olfactory disease: a map of Cochrane evidence relevant to rehabilitation for people with post COVID-19 condition. Eur J Phys Rehabil Med. 2022 Dec;58(6):875-879. doi:10.23736/S1973-9087.22.07811-X.  4. Han CH, Kim JH, Kim M, Kim HR, Kim SY, Choi HY, et al. Electroacupuncture for post-stroke dysphagia: a protocol for systematic review and meta-analysis of randomized controlled trials. Medicine (Baltimore). 2020 Sept 18;99(38):e22360.  5. Zhu R ran, Wang J xiang, Pan B, Lai H hao, Xu X ting, Ge L, et al. Evidence-based evaluation for stroke guidelines mentioning traditional and complementary medicine rehabilitation. BMC Complement Med Ther. 2025 May 19;25(1):184-96.  6. Wu M, Song W, Wang X, Tang Q, Gao W, Zhu L. Exploring the rules of related parameters in acupuncture for post-stroke dysphagia based on data mining. Front Neurol. 2024 May 24;15:1394348.  7. Qin W, Wang Z, Zhong Y, Yuan Q, Jiang X, Gao J, et al. Comparative efficacy of nonpharmaceutical therapy in the treatment of dysphagia after stroke: a protocol for systematic review. Medicine (Baltimore). 2020 Feb;99(9):e19115.  8. Jin C, Jang BH, Jeon JP, Lee YS, Yang SB, Kwon S. Traditional east asian herbal medicines for the treatment of poststroke constipation: a protocol for systematic review and meta analysis. Medicine (Baltimore). 2021 Apr 16;100(15):e25503. |
| Not dysphagia | 7 | 1.Yang ZX, Shi XM. Systematic review on the efficacy and safety of Xingnao Kaiqiao acupuncture for stroke [Chinese]. *Zhongguo Zhen Jiu*. 2007;27(8):601-8.  2.Dong Y, Tan LS, Liu J. Effects of eye acupuncture combined with ultra-early rehabilitation training on swallowing dysfunction and limb function recovery in patients with acute stroke [Chinese]. *Zhen Jiu Lin Chuang Za Zhi*. 2021;37(5):9-13.  3.Sun HY, Liu Y. Meta-analysis of acupuncture combined with swallowing rehabilitation training in the prevention of stroke-associated pneumonia [Chinese]. *Zhong Hua Zhen Jiu Dian Zi Za Zhi*. 2022;11(2):81-6.  4. Wu J, Wang Y, Wang X, Xie Y, Li W. A systematic review and meta-analysis of acupuncture in parkinson’s disease with dysphagia. Front Neurol. 2023 May 26;14:1099012.  5. Song S, Chen H. A systematic review and meta-analysis of randomized controlled trials for electroacupuncture treatment of migraine. J Pain Res. 2025 May;Volume 18:2321-33.  6. Zhao XF, Du Y, Liu PG, Wang S. Acupuncture for stroke: evidence of effectiveness, safety, and cost from systematic reviews. Top Stroke Rehabil. 2012 May;19(3):226-33.  7. Ang TE, Bivard A, Levi C, Ma H, Hsu CY, Campbell B, et al. Multi-modal CT in acute stroke: wait for a serum creatinine before giving intravenous contrast? No! Int J Stroke. 2015 Oct;10(7):1014-7. |
| Primary study | 7 | 1.Wang Y. Clinical efficacy evaluation of Mang-needle at Tiantu (CV22) in the treatment of true bulbar paralysis dysphagia after cerebral infarction: a randomized controlled study [Chinese] [Master's thesis]. Tianjin: Tianjin University of Traditional Chinese Medicine; 2023.  2.Li JA. Clinical study on the combination of acupuncture and rehabilitation for early hemiplegia, aphasia, and dysphagia of ischemic stroke [Chinese] [Dissertation]. Shijiazhuang: Hebei Medical University; 2005.  3.Hai R. Retrospective case study of rehabilitation treatment for Sa disease in the recovery stage [Chinese] [Master's thesis]. Tongliao: Inner Mongolia Minzu University; 2024.  4. Jiang ZF. Clinical study of "Tongguan Liqiao" acupuncture combined with Lianquan (CV23) and Tiantu (CV22) fast-needle deep-insertion for post-stroke dysphagia [Chinese] [Master's thesis]. Tianjin: Tianjin University of Traditional Chinese Medicine; 2023.  5.Lu YY. Literature and clinical study of acupuncture-movement therapy for post-stroke dysphagia [Chinese] [Dissertation]. Guangzhou: Guangzhou University of Chinese Medicine; 2021.  6.Mi SQ. Randomized controlled study of acupuncture and cupping as auxiliary treatment for pneumonia in the sequelae stage of stroke [Chinese] [Dissertation]. Beijing: Beijing University of Chinese Medicine; 2022.  7.Huang WX. Literature and clinical study of acupuncture combined with rehabilitation training for post-stroke dysphagia [Chinese] [Dissertation]. Guangzhou: Guangzhou University of Chinese Medicine; 2016. |
| Overview | 5 | 1.Fan WJ, Liang SB, Huang RY, et al. Overview of systematic reviews of acupuncture for post-stroke dysphagia [Chinese]. *Hai Nan Yi Xue Yuan Xue Bao*. 2020;26(11):829-37.  2.Liao WJ, Xu HY, Lyu YY. Overview of systematic reviews of acupuncture for post-stroke dysphagia in patients [Chinese]. *Dang Dai Hu Shi*. 2025;32(4):35-9.  3. Liu Z, Zhang Y, Yan X, Liu J. Acupuncture for stroke: an overview of systematic reviews. Integr Med Res. 2015 May;4(1):10-1.  4. Tian ZY, Liao X, Gao Y, Liang SB, Zhang CY, Xu DH, et al. An overview of systematic reviews and meta-analyses on acupuncture for post-acute stroke dysphagia. Geriatrics. 2019 Dec 8;4(4):68-85.  5. Zhang J huai, Wang D, Liu M. Overview of systematic reviews and meta-analyses of acupuncture for stroke. Neuroepidemiology. 2014;42(1):50-8. |
| Other ineligible type / insufficient info | 6 | 1. Zhou XN. Clinical evidence-based study on efficacy evaluation and optimal protocol selection of acupuncture for post-stroke dysphagia [Chinese] [Master's thesis]. Tianjin: Tianjin University of Traditional Chinese Medicine; 2016.  2.Xu JH. Study on the intervention of time-selected acupoint stimulation for patients with post-stroke dysphagia [Chinese] [Master's thesis]. Shijiazhuang: Hebei College of Traditional Chinese Medicine; 2021.  3.Guo WL. Clinical observation on the efficacy of acupuncture-movement therapy for post-stroke dysphagia in the recovery stage [Chinese] [Master's thesis]. Guangzhou: Guangzhou University of Chinese Medicine; 2025.  4.Liu H. Efficacy evaluation of acupuncture for dysphagia after stroke [Chinese] [Master's thesis]. Chengdu: Chengdu University of Traditional Chinese Medicine; 2016.  5.Sun JF. Clinical literature study of acupuncture for dysphagia after stroke [Chinese] [Master's thesis]. Shanghai: Shanghai University of Traditional Chinese Medicine; 2023.  6.Xie Y, Wang L, He J, et al. Acupuncture for dysphagia in acute stroke. Cochrane Database Syst Rev. 2008;(3):CD006076. doi:10.1002/14651858.CD006076.pub2 |
| Network meta-analysis | 6 | 1.Zheng Y. Network meta-analysis of non-pharmacological therapies for post-stroke dysphagia [Chinese] [Master's thesis]. Guangzhou: Guangzhou University of Chinese Medicine; 2021.  2.Li XY. Network meta-analysis of the efficacy of acupuncture for post-stroke dysphagia based on bibliometrics [Chinese] [Master's thesis]. Hangzhou: Zhejiang University of Chinese Medicine; 2025.  3.Hu JJ. Network meta-analysis of acupuncture combined with different electromagnetic stimulation protocols for improving post-stroke dysphagia [Chinese] [Master's thesis]. Wuhan: Hubei University of Chinese Medicine; 2025.  4.Xiong F, Song K, Huang A, et al. Treatment of dysphagia after stroke with acupuncture and related therapies: a protocol for systematic review and network meta-analysis. Medicine (Baltimore). 2020;99(34):e21657. doi:10.1097/MD.0000000000021657  5.Zhang B, Wong KP, Guo C, et al. Effects of non-pharmacological interventions on the swallowing function of patients with post-stroke dysphagia: a systematic review and network meta-analysis. J Oral Rehabil. 2025;52(1):109-20. doi:10.1111/joor.13824  6.Zhu H, Deng X, Luan G, et al. Comparison of efficacy of non-pharmacological intervention for post-stroke dysphagia: a systematic review and bayesian network meta-analysis. BMC Neurosci. 2023;24(1):53-66. doi:10.1186/s12868-023-00821-w |
| Health economic / evidence appraisal | 1 | 1.Zhang Y. Clinical evidence assessment and health economic evaluation of acupuncture for post-stroke dysphagia [Chinese] [Dissertation]. Guangzhou: Guangzhou University of Chinese Medicine; 2021. |
| Total | 76 |  |

**Table S4. Characteristics of included SRs/MAs**

Abbreviations: RCT, randomized controlled trial; SWAL-QOL, Swallowing Quality of Life; MDADI, M. D. Anderson Dysphagia Inventory; DSRS, Dysphagia Severity Rating Scale; FEES, Fiberoptic Endoscopic Evaluation of Swallowing; VFSS, Videofluoroscopic Swallowing Study; PAS, Penetration-Aspiration Scale.

Note. AMSTAR 2 overall confidence ratings are based on the original AMSTAR 2 critical-domain configuration, which is retained as the reference assessment.

| **Study ID** | **Review** | **Country/region** | **Protocol registration** | **Included RCTs  (n)** | **Participants  (n)** | **Acupuncture type** | **Comparator type** | **Primary outcome domain** | **Main instruments** | **AMSTAR 2 overall** | **Overall conclusion** |
| --- | --- | --- | --- | --- | --- | --- | --- | --- | --- | --- | --- |
| P01 | Zhang Shaohua, 2017 | China | Not reported | 11 | 735 | Electroacupuncture | Head-to-head acupuncture comparator | Effective rate | VFSS; WST | Critically low | Probably beneficial |
| P02 | Zhang Yi, 2017 | China | Not reported | 12 | 824 | Electroacupuncture | Head-to-head acupuncture comparator | Effective rate | VFSS; WST | Critically low | Probably beneficial |
| P03 | Geng Jiujun, 2021 | China | Not reported | 18 | 1352 | Electroacupuncture | Add-on design with conventional treatment / rehabilitation control | Effective rate | FILS; VFSS; WST | Critically low | Probably beneficial |
| P04 | Zhu Yuan, 2012 | China | Not reported | 4 | 701 | Mixed (electroacupuncture + manual body acupuncture) | Add-on design with conventional treatment / rehabilitation control | Effective rate | WST | Critically low | Probably beneficial |
| P05 | Wang Jinping, 2023 | China | Not reported | 12 | 690 | Intradermal/press/embedded needles | Add-on design with conventional treatment / rehabilitation control | Swallowing function scale | SSA; SWAL-QOL/QoL; WST | Critically low | Probably beneficial |
| P06 | Yang Jiwei, 2018 | China | Not reported | 8 | 667 | Tongue acupuncture | Conventional treatment / rehabilitation control | Effective rate | FILS; WST | Critically low | Probably beneficial |
| P07 | Xia Yuge, 2021 | China | Not reported | 21 | 1532 | Three-tongue-needle / tongue acupuncture | Mixed comparators (rehabilitation / medication / acupuncture / devices) | Effective rate | SSA; WST | Critically low | Probably beneficial |
| P08 | Hu Shiying, 2021 | China | Not reported | 9 | 671 | Three-tongue-needle / tongue acupuncture | Add-on design with conventional treatment / rehabilitation control | Effective rate | VFSS; WST | Critically low | Probably beneficial |
| P09 | Xu Hong, 2024 | China | Not reported | 11 | 1006 | Tongdu Tiaoshen acupuncture (manual ± electroacupuncture) | Mixed comparators (rehabilitation / swallowing training / conventional acupuncture / feeding training / devices) | Effective rate | VFSS; SSA; WST | Critically low | Probably beneficial |
| P10 | Hu Tianjun, 2015 | China | Not reported | 17 | 1158 | Manual/traditional body acupuncture | Conventional treatment / rehabilitation control | Effective rate | NR | Critically low | Probably beneficial |
| P11 | Zhao Nan, 2021 | China | Not reported | 19 | 1874 | Manual/traditional body acupuncture | Head-to-head acupuncture comparator | Swallowing function scale | SSA; SWAL-QOL/QoL; WST | Critically low | Probably beneficial |
| P12 | Chen Jie, 2015 | China | Not reported | 8 | 766 | Mixed (multiple acupuncture modalities) | Mixed comparators (medical care / swallowing training / conventional acupuncture) | Effective rate | WST; SSA; FILS | Critically low | Probably beneficial |
| P13 | Xu Lianjie, 2019 | China | Not reported | 22 | 1998 | Manual/traditional body acupuncture | Head-to-head acupuncture comparator | Effective rate | WST; SSA; FILS | Critically low | Probably beneficial |
| P14 | Sun Hongqiong, 2024 | China | Not reported | 28 | 2879 | Xingnao Kaiqiao acupuncture | Mixed comparators (non-Xingnao Kaiqiao therapies) | Effective rate | VFSS; SSA; WST; SWAL-QOL/QoL | Critically low | Probably beneficial |
| P15 | Si Huijie, 2023 | China | Not reported | 15 | 1205 | Ocular acupuncture | Head-to-head acupuncture comparator | Swallowing function scale | VFSS; WST | Critically low | Probably beneficial |
| P16 | Li Tingting, 2021 | China | Not reported | 30 | 2446 | Mixed (electroacupuncture + manual body acupuncture) | Conventional treatment / rehabilitation control | Aspiration / pneumonia | SSA; WST | Critically low | Probably beneficial |
| P17 | Yuan Menglang, 2011 | China | Not reported | 13 | 962 | Manual/traditional body acupuncture | Add-on design with conventional treatment / rehabilitation control | Effective rate | NR | Critically low | Probably beneficial |
| P18 | Tang Qiang, 2019 | China | Not reported | 22 | 1987 | Manual/traditional body acupuncture | Sham acupuncture | Instrumental assessment | VFSS; SSA; WST | Critically low | Probably beneficial |
| P19 | Wang Huan, 2019 | China | Not reported | 12 | 921 | Manual/traditional body acupuncture | Add-on design with conventional treatment / rehabilitation control | Swallowing function scale | SSA; SWAL-QOL | Critically low | Probably beneficial |
| P20 | Tang Xiaorong, 2019 | China | Not reported | 16 | 1780 | Manual/traditional body acupuncture | Add-on design with conventional treatment / rehabilitation control | Swallowing function scale | Clinical dysphagia scale | Low | Probably beneficial |
| P21 | Tan Shiyun, 2020 | China | Not reported | 12 | 903 | Manual/traditional body acupuncture | Head-to-head acupuncture comparator | Instrumental assessment | VFSS; WST | Low | Probably beneficial |
| P22 | Zuo Tianxiang, 2023 | China | Not reported | 15 | 859 | Manual/traditional body acupuncture | Add-on design with conventional treatment / rehabilitation control | Instrumental assessment | VFSS; SSA; PAS; FOIS | Critically low | Probably beneficial |
| P23 | Xu Minghua, 2017 | China | Not reported | 11 | 907 | Manual/traditional body acupuncture | Non-acupuncture conventional control (rehabilitation / electrical stimulation / herbal medicine, etc.) | Effective rate | NR | Critically low | Probably beneficial |
| P24 | Zhao Dongfeng, 2019 | China | Not reported | 28 | 2557 | Mixed (electroacupuncture + scalp acupuncture + tongue acupuncture + manual body acupuncture) | Sham acupuncture | Aspiration / pneumonia | MBI; SWAL-QOL | Low | Probably beneficial |
| P25 | Wang Chun, 2017 | China | Not reported | 32 | 1763 | Manual/traditional body acupuncture | Add-on design with conventional treatment / rehabilitation control | Effective rate | NR | Critically low | Probably beneficial |
| P26 | Wang Jie, 2021 | China | Not reported | 14 | 954 | Manual/traditional body acupuncture | Add-on design with conventional treatment / rehabilitation control | Swallowing function scale | WST | Critically low | Probably beneficial |
| P27 | Yu Chuan, 2016 | China | Not reported | 9 | 577 | Manual/traditional body acupuncture | Add-on design with conventional treatment / rehabilitation control | Swallowing function scale | WST | Low | Probably beneficial |
| P28 | Wang Liping, 2006 | China | Not reported | 7 | 506 | Mixed (electroacupuncture + manual body acupuncture) | No treatment / blank control | Swallowing function scale | Swallowing function scale | Critically low | Inconclusive |
| P29 | Feng Ye, 2020 | China | Not reported | 32 | 3719 | Electroacupuncture | No treatment / blank control | Swallowing function scale | SSA; WST | Critically low | Probably beneficial |
| P30 | Wang Li, 2024 | China | Not reported | 22 | 1816 | Manual/traditional body acupuncture | Head-to-head acupuncture comparator | Instrumental assessment | VFSS; WST | Critically low | Probably beneficial |
| P31 | Yihe Tang, 2022 | China | PROSPERO (CRD42021230472) | 11 | 1069 | Manual/traditional body acupuncture | Add-on design with conventional treatment / rehabilitation control | Instrumental assessment | VFSS; SSA; FEES | Critically low | Probably beneficial |
| P32 | Yao-Bin Long, 2012 | China | Not reported | 72 | 6134 | Manual/traditional body acupuncture | Add-on design with conventional treatment / rehabilitation control | Effective rate | NR | Critically low | Probably beneficial |
| P33 | Jing Luo, 2023 | China | PROSPERO (CRD42022350411) | 10 | 517 | Mixed (manual body acupuncture + electroacupuncture + tongue acupuncture) | Balloon dilation control (device/procedure) | Instrumental assessment | VFSS; SSA | Critically low | Probably beneficial |
| P34 | Ling Xin Li, 2019 | Unclear (likely China; all included studies from China) | Not reported | 17 | 1479 | Manual/traditional body acupuncture | Add-on design with conventional treatment / rehabilitation control | Instrumental assessment | VFSS; SSA; FDS; MBI | Critically low | Probably beneficial |
| P35 | Ivy S.Y. Wong, 2012 | Hong Kong, China | Not reported | 9 | 864 | Mixed (electroacupuncture + manual body acupuncture) | Add-on design with conventional treatment / rehabilitation control | Instrumental assessment | VFSS; SSA; SWAL-QOL | Critically low | Probably beneficial |
| P36 | LI Ling-xin, 2018 | China | Not reported | 29 | 2190 | Mixed (electroacupuncture + manual body acupuncture) | Sham acupuncture | Instrumental assessment | VFSS; SSA; WST | Critically low | Probably beneficial |
| P37 | Jinke Huang, 2020 | China | Not reported | 16 | 1216 | Electroacupuncture | Add-on design with conventional treatment / rehabilitation control | Instrumental assessment | VFSS; WST; IFRS | Critically low | Probably beneficial |
| P38 | Jiongliang Zhang, 2025 | China | CRD42024613132 | 20 | 1718 | Mixed (scalp acupuncture + tongue acupuncture + manual body acupuncture) | Add-on design with conventional treatment / rehabilitation control | Instrumental assessment | VFSS; SSA; WST; SWAL-QOL | Moderate | Probably beneficial |
| P39 | Wang P, 2022 | China | PROSPERO (CRD42022306827) | 7 | 637 | Manual/traditional body acupuncture | Add-on design with conventional treatment / rehabilitation control | Instrumental assessment | VFSS; SSA | Low | Probably beneficial |
| P40 | Yanyan Lu, 2021 | China | Not reported | 39 | 3078 | Manual/traditional body acupuncture | Add-on design with conventional treatment / rehabilitation control | Instrumental assessment | VFSS; SSA; WST; SWAL-QOL; Fujishima feeding-swallowing scale | Critically low | Probably beneficial |
| P41 | Hailun Jiang, 2022 | China | PROSPERO (CRD42021258346) | 33 | 2680 | Manual/traditional body acupuncture | Add-on design with conventional treatment / rehabilitation control | Instrumental assessment | VFSS; SSA; WST; BI; SWAL-QOL | Low | Probably beneficial |
| P42 | Xuezheng Li, 2023 | China | PROSPERO (CRD42023396840) | 12 | 1358 | Electroacupuncture | Mixed comparators (rehabilitation / medication / acupuncture / devices) | Instrumental assessment | VFSS; SSA; PAS; SWAL-QOL | Low | Probably beneficial |
| P43 | Qiuping Ye, 2017 | China | Not reported | 71 | 6010 | Manual/traditional body acupuncture | No treatment / blank control | Swallowing function scale | SSA; WST; VFSS; ADL; SWAL-QOL; IFRS | Critically low | Probably beneficial |
| P44 | Lida Zhong, 2021 | China | INPLASY (2020100036) | 35 | 3024 | Mixed (electroacupuncture + manual body acupuncture) | Add-on design with conventional treatment / rehabilitation control | Instrumental assessment | VFSS; SSA; WST; IFRS; BI; SWAL-QOL; sEMG | Low | Probably beneficial |
| P45 | Jing Luo, 2022 | China | Not reported | 15 | 1094 | Mixed (scalp acupuncture + tongue acupuncture + manual body acupuncture) | Head-to-head acupuncture comparator | Instrumental assessment | VFSS; SSA; WST | Critically low | Probably beneficial |

Table S5. Overlap analysis details and CCA calculation

| **Parameter** | **Value** | **Description** |
| --- | --- | --- |
| **Total Citations (N)** | **894** | Total count of RCT citations extracted from 45 SRs (after excluding non-RCTs). |
| **Unique Primary Studies (c)** | **779** | Count of unique RCTs after deduplication. |
| **Number of Reviews (r)** | **45** | Number of included systematic reviews. |
| **CCA Formula** | (N−c)/(c×(r−1)) | Formula based on Pieper et al. (2014). |
| **CCA Result** | **0.34%** | Calculated overlap value. |
| **Interpretation** | **Slight Overlap** | According to Pieper's threshold (<5%). |

Table S6A. Item-level AMSTAR 2 assessment of included systematic reviews/meta-analyses

| **Study ID** | **Item 1** | **Item 2** | **Item 3** | **Item 4** | **Item 5** | **Item 6** | **Item 7** | **Item 8** | **Item 9** | **Item 10** | **Item 11** | **Item 12** | **Item 13** | **Item 14** | **Item 15** | **Item 16** |
| --- | --- | --- | --- | --- | --- | --- | --- | --- | --- | --- | --- | --- | --- | --- | --- | --- |
| Zhang SH/2017 | Y | N | N | P | Y | Y | N | P | Y | N | No | No | N | N | No | N |
| Zhang Y/2017 | Y | N | N | P | Y | Y | N | Y | Y | N | Y | N | Y | Y | Y | N |
| Geng JJ/2021 | Y | N | N | P | Y | Y | N | Y | Y | N | Y | N | Y | Y | Y | N |
| Zhu Y/2012 | Y | N | N | P | Y | Y | N | P | Y | N | Y | Y | Y | Y | Y | N |
| Wang JP/2023 | Y | N | N | Y | Y | Y | N | P | Y | N | Y | Y | Y | Y | Y | N |
| Yang JW/2018 | Y | N | N | P | N | N | N | P | Y | N | Y | Y | Y | Y | Y | N |
| Xia YG/2021 | Y | N | N | P | Y | Y | N | P | Y | N | No | No | Y | Y | No | N |
| Hu SY/2021 | Y | N | N | P | Y | Y | N | P | Y | N | Y | Y | Y | Y | Y | N |
| Xu H/2024 | Y | N | N | P | Y | Y | N | Y | N | Y | Y | Y | Y | Y | Y | N |
| Hu TJ/2015 | Y | N | N | P | Y | Y | N | Y | Y | N | Y | Y | Y | Y | Y | N |
| Zhao N/2021 | Y | N | N | P | Y | Y | N | P | Y | N | Y | N | N | Y | Y | N |
| Chen J/2015 | Y | N | N | Y | Y | Y | N | Y | Y | N | No | No | N | Y | No | N |
| Xu LJ/2019 | Y | N | N | P | Y | Y | N | N | Y | N | Y | Y | Y | Y | Y | N |
| Sun HJ/2024 | Y | N | N | P | Y | Y | N | P | Y | N | Y | N | Y | Y | Y | N |
| Si HJ/2023 | Y | N | N | Y | Y | Y | N | Y | Y | N | Y | Y | Y | Y | Y | N |
| Li TT/2021 | Y | N | N | Y | Y | Y | N | Y | Y | N | Y | Y | Y | Y | Y | N |
| Yuan ML/2011 | Y | N | N | P | Y | Y | N | P | Y | N | Y | Y | Y | Y | Y | N |
| Tang Q/2019 | Y | N | N | Y | Y | Y | N | Y | Y | N | Y | Y | Y | Y | Y | N |
| Wang H/2019 | Y | N | N | Y | Y | Y | N | Y | Y | N | Y | N | Y | Y | Y | N |
| Tang XR/2019 | Y | N | N | P | Y | Y | Y | P | Y | N | Y | Y | Y | Y | Y | N |
| Tan SY/2020 | Y | N | N | Y | Y | N | Y | Y | Y | N | Y | Y | Y | Y | Y | N |
| Zuo TX/2023 | Y | N | N | P | Y | Y | P | Y | Y | N | Y | Y | N | Y | N | N |
| Xu MH/2017 | Y | N | N | P | Y | Y | P | Y | Y | N | N | N | N | N | N | N |
| Zhao DF/2019 | Y | N | N | Y | Y | Y | Y | Y | Y | N | Y | Y | Y | N | Y | N |
| Wang C/2017 | Y | N | N | Y | Y | Y | N | P | Y | N | Y | N | Y | Y | Y | N |
| Wang J/2021 | Y | N | N | Y | Y | Y | N | Y | Y | N | Y | Y | Y | Y | Y | N |
| Yu C/2016 | Y | N | N | Y | Y | Y | P | Y | Y | N | Y | N | Y | N | Y | N |
| Wang LP/2006 | Y | N | N | Y | Y | Y | N | Y | Y | N | Y | N | N | N | Y | N |
| Feng Y/2020 | Y | N | N | P | Y | Y | N | P | Y | N | Y | N | Y | N | Y | N |
| Wang L/2024 | Y | N | N | Y | Y | Y | N | Y | Y | N | Y | Y | Y | Y | Y | N |
| Yihe Tang/2022 | Y | Y | N | Y | Y | Y | N | Y | Y | N | Y | Y | Y | Y | N | Y |
| LingXin Li/2019 | Y | N | Y | Y | Y | Y | N | Y | Y | N | Y | N | Y | Y | Y | Y |
| Jing Luo/2023 | Y | Y | N | Y | Y | Y | N | Y | Y | N | Y | Y | Y | Y | N | Y |
| Yaobin Long/2012 | Y | N | N | Y | Y | Y | N | Y | Y | N | Y | N | Y | N | N | Y |
| Wong /2012 | Y | N | Y | Y | Y | Y | N | Y | Y | N | No | No | Y | Y | No | N |
| Lingxin Li/2018 | Y | N | Y | Y | Y | Y | N | Y | Y | N | Y | Y | Y | Y | Y | Y |
| Jinke Huang/2020 | Y | N | N | Y | Y | Y | N | Y | Y | N | Y | Y | Y | Y | Y | Y |
| Jiongliang Zhang/2025 | Y | Y | N | Y | Y | Y | Y | Y | Y | N | Y | Y | Y | Y | Y | Y |
| Peng Wang/2021 | Y | Y | N | Y | Y | Y | N | Y | Y | N | Y | Y | Y | Y | Y | Y |
| Yanyan Lu/2021 | Y | N | N | P | Y | Y | N | Y | Y | N | Y | N | Y | Y | Y | Y |
| Hailun Jiang/2022 | Y | Y | Y | Y | Y | Y | N | Y | Y | N | Y | Y | Y | Y | Y | Y |
| Xuezheng Li/2023 | Y | Y | N | Y | Y | Y | N | Y | Y | N | Y | Y | Y | Y | Y | Y |
| Qiuping Ye/2017 | Y | N | N | Y | Y | Y | N | Y | Y | N | Y | N | Y | Y | N | Y |
| Lida Zhong/2021 | Y | Y | N | Y | Y | Y | N | Y | Y | N | Y | Y | Y | Y | Y | Y |
| Li Li/2023 | Y | N | N | Y | Y | Y | N | Y | Y | N | Y | Y | Y | Y | Y | Y |

****Item-level AMSTAR 2 ratings are presented for all included systematic reviews/meta-analyses (SRs/MAs). Ratings were coded as Y (Yes), P (Partial Yes), and N (No) according to the AMSTAR 2 guidance. Overall confidence ratings were evaluated using two configurations: the original AMSTAR 2 critical-domain configuration and an alternative configuration in which Item 7 was not considered a critical domain. Study IDs are reported in the format of first author/year for consistency with the main text and other supplementary materials.****

****Table S6B. Overall confidence distribution of AMSTAR 2 ratings under the two critical-domain configurations****

| **Overall confidence distribution** |  |  |  |
| --- | --- | --- | --- |
| Rating level | Alternative configuration (Item 7 non-critical) | Original AMSTAR 2 configuration (Item 7 critical) | Difference |
| High | 0 | 0 | 0 |
| Moderate | 5 | 1 | -4 |
| Low | 30 | 8 | -22 |
| Critically low | 10 | 36 | 26 |

**Table S6C. Study-level comparison of AMSTAR 2 overall confidence ratings under the two critical-domain configurations**

Note. Study IDs correspond to Supplementary Table S4. The original AMSTAR 2 critical-domain configuration was retained as the reference assessment. The alternative configuration differs only in that Item 7 is not treated as a critical domain. Item 7 ratings are coded as Y (Yes), P (Partial Yes), and N (No).

| **No.** | **Study ID** | **Item 7 rating** | **Alternative configuration (Item 7 non-critical)** | **Original AMSTAR 2 configuration (Item 7 critical)** |
| --- | --- | --- | --- | --- |
| 1 | P01 | N | Critically low | Critically low |
| 2 | P02 | N | Low | Critically low |
| 3 | P03 | N | Low | Critically low |
| 4 | P04 | N | Low | Critically low |
| 5 | P05 | N | Low | Critically low |
| 6 | P06 | N | Low | Critically low |
| 7 | P07 | N | Low | Critically low |
| 8 | P08 | N | Low | Critically low |
| 9 | P09 | N | Critically low | Critically low |
| 10 | P10 | N | Low | Critically low |
| 11 | P11 | N | Critically low | Critically low |
| 12 | P12 | N | Critically low | Critically low |
| 13 | P13 | N | Low | Critically low |
| 14 | P14 | N | Low | Critically low |
| 15 | P15 | N | Low | Critically low |
| 16 | P16 | N | Low | Critically low |
| 17 | P17 | N | Low | Critically low |
| 18 | P18 | N | Low | Critically low |
| 19 | P19 | N | Low | Critically low |
| 20 | P20 | Y | Low | Low |
| 21 | P21 | Y | Low | Low |
| 22 | P22 | P | Critically low | Critically low |
| 23 | P23 | P | Critically low | Critically low |
| 24 | P24 | Y | Low | Low |
| 25 | P25 | N | Low | Critically low |
| 26 | P26 | N | Low | Critically low |
| 27 | P27 | P | Low | Low |
| 28 | P28 | N | Critically low | Critically low |
| 29 | P29 | N | Low | Critically low |
| 30 | P30 | N | Low | Critically low |
| 31 | P31 | N | Low | Critically low |
| 32 | P32 | N | Low | Critically low |
| 33 | P33 | N | Critically low | Critically low |
| 34 | P34 | N | Critically low | Critically low |
| 35 | P35 | N | Low | Critically low |
| 36 | P36 | N | Low | Critically low |
| 37 | P37 | N | Low | Critically low |
| 38 | P38 | Y | Moderate | Moderate |
| 39 | P39 | N | Moderate | Low |
| 40 | P40 | N | Low | Critically low |
| 41 | P41 | N | Moderate | Low |
| 42 | P42 | N | Moderate | Low |
| 43 | P43 | N | Critically low | Critically low |
| 44 | P44 | N | Moderate | Low |
| 45 | P45 | N | Low | Critically low |

Table S7. GRADE certainty of evidence for included outcomes

Note. GRADE ratings were assessed at the outcome level. 0 = no downgrading; -1 = downgraded by one level; -2 = downgraded by two levels. Figure 3 in the main text summarizes outcome-domain coverage at the review level; therefore, the counts in the supplementary GRADE tables are not expected to match exactly. No GRADE table was provided for nutrition outcomes because this domain was not reported in any included SR/MA.

Table S7A. GRADE certainty of evidence for effective rate and related composite response outcomes

| **Study** | **Intervention/comparison** | **Specific outcome** | **No. of RCTs**  **(participants)** | **Effect estimate**  **(95% CI)** | **RoB** | **Inconsistency** | **Indirectness** | **Imprecision** | **Publication bias** | **Quality of evidence** | **Main reasons for downgrading** |
| --- | --- | --- | --- | --- | --- | --- | --- | --- | --- | --- | --- |
| Wang LP (2006) | Acupuncture alone or acupuncture + conventional care vs non-acupuncture therapy (swallowing training / rehabilitation / medication) | Total effective rate | 6 RCTs (n = 436) | RR 1.17, 95% CI 1.08 to 1.27 | Very serious | Not serious | Not serious | Not serious | Not serious | Low | Risk of bias |
| Yuan ML (2011) | Acupuncture + rehabilitation training vs rehabilitation or other non-combined treatment | Cure rate | 13 RCTs (n = 962) | OR 3.04, 95% CI 2.29 to 4.06 | Very serious | Not serious | Serious | Not serious | Not serious | Very low | Risk of bias; Indirectness |
| Yuan ML (2011) | Acupuncture + rehabilitation training vs rehabilitation or other non-combined treatment | Total effective rate | 13 RCTs (n = 962) | OR 5.39, 95% CI 3.93 to 7.39 | Very serious | Not serious | Serious | Not serious | Not serious | Very low | Risk of bias; Indirectness |
| Long YB (2012) | Acupuncture + medicine and/or rehabilitation vs medicine and/or rehabilitation without acupuncture | Total effective rate | 72 RCTs (n = 6,134) | OR 5.17, 95% CI 4.18 to 6.38 | Serious | Not serious | Serious | Not serious | Not serious | Low | Risk of bias; Indirectness |
| Zhu Y (2012) | Acupuncture or acupuncture + rehabilitation vs rehabilitation | Cure rate | 5 RCTs (n = 517) | RR 2.86, 95% CI 1.82 to 4.50 | Very serious | Serious | Serious | Not serious | Serious | Very low | Risk of bias; Inconsistency; Indirectness; Publication bias |
| Zhu Y (2012) | Acupuncture or acupuncture + rehabilitation vs rehabilitation | Total effective rate | 7 RCTs (n = 701) | RR 1.39, 95% CI 1.15 to 1.69 | Very serious | Very serious | Serious | Not serious | Serious | Very low | Risk of bias; Inconsistency; Indirectness; Publication bias |
| Chen J (2015) | Xingnao Kaiqiao acupuncture + usual care vs usual care, recovery stage, 4-week treatment | Total effective rate | 3 RCTs (n = 313) | RR 1.35, 95% CI 1.09 to 1.68 | Very serious | Not serious | Serious | Not serious | Serious | Very low | Risk of bias; Indirectness; Publication bias |
| Chen J (2015) | Xingnao Kaiqiao acupuncture + usual care vs usual care, acute stage, 4-week treatment | Total effective rate | 2 RCTs (n = 290) | RR 1.39, 95% CI 1.00 to 1.94 | Very serious | Very serious | Serious | Serious | Serious | Very low | Risk of bias; Inconsistency; Indirectness; Imprecision; Publication bias |
| Hu TJ (2015) | Nape acupuncture alone or nape acupuncture + co-intervention vs non-nape acupuncture control | Cure rate | 17 RCTs (n = 1,158) | OR 2.67, 95% CI 2.03 to 3.53 | Serious | Not serious | Serious | Not serious | Not serious | Low | Risk of bias; Indirectness |
| Hu TJ (2015) | Nape acupuncture alone or nape acupuncture + co-intervention vs non-nape acupuncture control | Total effective rate | 17 RCTs (n = 1,158) | OR 3.99, 95% CI 2.83 to 5.63 | Serious | Not serious | Serious | Not serious | Not serious | Low | Risk of bias; Indirectness |
| Yu C (2016) | Acupuncture alone or acupuncture + swallowing rehabilitation vs swallowing rehabilitation without acupuncture | Total effective rate | 9 RCTs (n = 1,017) | RR 0.36, 95% CI 0.25 to 0.50 | Very serious | Not serious | Not serious | Not serious | Not serious | Low | Risk of bias |
| Wang C (2017) | Acupuncture + western medicine vs western medicine | Total effective rate | 11 RCTs (n = 973) | OR 5.78, 95% CI 4.03 to 8.27 | Serious | Not serious | Serious | Not serious | Not serious | Low | Risk of bias; Indirectness |
| Wang C (2017) | Acupuncture + rehabilitation vs rehabilitation | Total effective rate | 19 RCTs (n = 1,678) | OR 3.84, 95% CI 2.91 to 5.05 | Serious | Not serious | Serious | Not serious | Not serious | Low | Risk of bias; Indirectness |
| Xu MH (2017) | Acupuncture therapy vs non-acupuncture therapies (rehabilitation training, swallowing therapy, electrical stimulation, oral herbal medicine, etc.) | Total effective rate | 11 RCTs (n = 907) | OR 3.97, 95% CI 2.73 to 5.76 | Very serious | Not serious | Not serious | Not serious | Not serious | Low | Risk of bias |
| Ye QP (2017) | Acupuncture alone or acupuncture combined with co-interventions vs rehabilitation / medication / sham or other non-acupuncture controls | Clinical efficacy rate | 58 RCTs (n not clearly reported) | RR 1.17, 95% CI 1.13 to 1.21 | Very serious | Serious | Serious | Not serious | Not serious | Very low | Risk of bias; Inconsistency; Indirectness |
| Ye QP (2017) | Acupuncture alone or acupuncture combined with co-interventions vs rehabilitation / medication / sham or other non-acupuncture controls | WST efficacy rate | 9 RCTs (n not clearly reported) | RR 1.25, 95% CI 1.03 to 1.50 | Very serious | Serious | Serious | Not serious | Not serious | Very low | Risk of bias; Inconsistency; Indirectness |
| Zhang SH (2017) | Electroacupuncture or electroacupuncture + swallowing training vs control | Total effective rate | 11 RCTs (n = 735) | OR 5.37, 95% CI 3.57 to 8.07 | Very serious | Not serious | Serious | Not serious | Serious | Very low | Risk of bias; Indirectness; Publication bias |
| Zhang Y (2017) | Electroacupuncture or electroacupuncture + co-intervention vs control | Markedly effective rate | 12 RCTs (n = 824) | RR 1.68, 95% CI 1.39 to 2.02 | Very serious | Serious | Serious | Not serious | Serious | Very low | Risk of bias; Inconsistency; Indirectness; Publication bias |
| Zhang Y (2017) | Electroacupuncture or electroacupuncture + co-intervention vs control | Total effective rate | 12 RCTs (n = 824) | RR 1.34, 95% CI 1.25 to 1.44 | Very serious | Not serious | Serious | Not serious | Serious | Very low | Risk of bias; Indirectness; Publication bias |
| Li LX (2018) | Acupuncture vs non-acupuncture treatment (rehabilitation and/or routine medication) | Overall effective rate of dysphagia improvement | 29 RCTs (n = 2,190) | RR 1.33, 95% CI 1.25 to 1.43 | Serious | Serious | Serious | Not serious | Serious | Very low | Risk of bias; Inconsistency; Indirectness; Publication bias |
| Yang JW (2018) | Tongue tri-points therapy or tongue tri-points + co-intervention vs control | Clinical effective rate | 8 RCTs (n = 667) | OR 5.37, 95% CI 3.20 to 9.01 | Serious | Not serious | Serious | Not serious | Not serious | Low | Risk of bias; Indirectness |
| Li LX (2019) | Acupuncture + swallowing training vs swallowing training alone | Effective rate | 14 RCTs (n not clearly reported) | RR 1.26, 95% CI 1.19 to 1.34 | Serious | Not serious | Not serious | Not serious | Serious | Low | Risk of bias; Publication bias |
| Tang Q (2019) | Acupuncture + rehabilitation training vs rehabilitation training / sham acupuncture + rehabilitation | Total effective rate | 17 RCTs (n = 1,601) | OR 3.66, 95% CI 2.66 to 5.05 | Serious | Not serious | Serious | Not serious | Serious | Very low | Risk of bias; Indirectness; Publication bias |
| Tang XR (2019) | Acupuncture + rehabilitation therapy vs rehabilitation alone and/or conventional western medicine | Total effective rate | 13 RCTs (n = 1,511) | RR 1.22, 95% CI 1.16 to 1.28 | Serious | Serious | Serious | Not serious | Serious | Very low | Risk of bias; Inconsistency; Indirectness; Publication bias |
| Wang H (2019) | Acupuncture + rehabilitation training vs swallowing training or routine treatment | Clinical efficacy | 8 RCTs (n = 651) | RR 3.64, 95% CI 2.31 to 5.73 | Serious | Not serious | Not serious | Not serious | Serious | Low | Risk of bias; Publication bias |
| Xu LJ (2019) | Xingnao Kaiqiao acupuncture alone or combined with co-intervention vs non-Xingnao Kaiqiao control | Cure rate | 12 RCTs (n = 1,019) | OR 3.83, 95% CI 2.77 to 5.29 | Very serious | Not serious | Not serious | Not serious | Not serious | Low | Risk of bias |
| Xu LJ (2019) | Xingnao Kaiqiao acupuncture alone or combined with co-intervention vs non-Xingnao Kaiqiao control | Total effective rate | 22 RCTs (n = 1,998) | OR 4.61, 95% CI 3.51 to 6.04 | Very serious | Not serious | Not serious | Not serious | Not serious | Low | Risk of bias |
| Zhao DF (2019) | Acupuncture-based treatment (alone or combined with rehabilitation/drugs) vs non-acupuncture regular care | Total effective rate | 28 RCTs (n = 2,557) | OR 4.11, 95% CI 3.29 to 5.13 | Serious | Not serious | Serious | Not serious | Serious | Very low | Risk of bias; Indirectness; Publication bias |
| Feng Y (2020) | Acupuncture + TCM vs acupuncture | Total effective rate | 9 RCTs (n not clearly reported) | RR 1.23, 95% CI 1.17 to 1.30 | Serious | Not serious | Serious | Not serious | Not serious | Low | Risk of bias; Indirectness |
| Feng Y (2020) | Acupuncture + TCM vs TCM alone | Total effective rate | 4 RCTs (n not clearly reported) | RR 1.36, 95% CI 1.21 to 1.52 | Serious | Not serious | Serious | Not serious | Not serious | Low | Risk of bias; Indirectness |
| Feng Y (2020) | Acupuncture + TCM vs western medicine | Total effective rate | 2 RCTs (n not clearly reported) | RR 1.16, 95% CI 1.06 to 1.27 | Serious | Not serious | Serious | Not serious | Not serious | Low | Risk of bias; Indirectness |
| Feng Y (2020) | Acupuncture + TCM vs blank/control treatment | Total effective rate | 17 RCTs (n not clearly reported) | RR 1.20, 95% CI 1.16 to 1.24 | Serious | Not serious | Serious | Not serious | Not serious | Low | Risk of bias; Indirectness |
| Huang J (2020) | Electroacupuncture + swallowing rehabilitation training vs swallowing rehabilitation training alone | Effective rate | 12 RCTs (n = 968) | OR 5.40, 95% CI 3.78 to 7.72 | Serious | Not serious | Serious | Not serious | Not serious | Low | Risk of bias; Indirectness |
| Tan SY (2020) | Acupuncture + swallowing function training vs conventional treatment / swallowing training | Clinical total effective rate | 12 RCTs (n = 903) | RR 1.22, 95% CI 1.15 to 1.29 | Serious | Not serious | Not serious | Not serious | Serious | Low | Risk of bias; Publication bias |
| Geng JJ (2021) | Electroacupuncture + swallowing training vs swallowing training | Total effective rate | 14 RCTs (n = 1,034) | OR 4.87, 95% CI 3.47 to 6.83 | Serious | Not serious | Serious | Not serious | Not serious | Low | Risk of bias; Indirectness |
| Hu SY (2021) | Tongue three-needle alone or tongue three-needle + co-intervention vs non-tongue three-needle control | Cure rate | 6 RCTs (n = 504) | OR 2.68, 95% CI 1.78 to 4.04 | Serious | Not serious | Serious | Not serious | Not serious | Low | Risk of bias; Indirectness |
| Hu SY (2021) | Tongue three-needle alone or tongue three-needle + co-intervention vs non-tongue three-needle control | Total effective rate | 9 RCTs (n = 731) | OR 4.01, 95% CI 2.58 to 6.25 | Serious | Not serious | Serious | Not serious | Not serious | Low | Risk of bias; Indirectness |
| Li (2021) | Electroacupuncture vs control | Markedly effective rate | 3 RCTs (n = 179) | RR 2.25, 95% CI 1.49 to 3.40 | Serious | Not serious | Not serious | Not serious | Serious | Low | Risk of bias; Publication bias |
| Li (2021) | Acupuncture vs control | Markedly effective rate | 27 RCTs (n = 2,068) | RR 1.45, 95% CI 1.34 to 1.58 | Serious | Not serious | Not serious | Not serious | Serious | Low | Risk of bias; Publication bias |
| Li (2021) | Electroacupuncture vs control | Total effective rate | 3 RCTs (n = 179) | RR 1.53, 95% CI 1.14 to 2.06 | Serious | Serious | Not serious | Not serious | Serious | Very low | Risk of bias; Inconsistency; Publication bias |
| Li (2021) | Acupuncture vs control | Total effective rate | 27 RCTs (n = 2,258) | RR 1.21, 95% CI 1.16 to 1.25 | Serious | Not serious | Not serious | Not serious | Serious | Low | Risk of bias; Publication bias |
| Lu Y (2021) | Acupuncture + swallowing rehabilitation training vs swallowing rehabilitation training | Effective rate | 36 RCTs (n = 2,846) | RR 1.23, 95% CI 1.19 to 1.27 | Serious | Not serious | Serious | Not serious | Not serious | Low | Risk of bias; Indirectness |
| Wang J (2021) | Acupuncture + swallowing training vs conventional rehabilitation / swallowing training | Clinical marked effectiveness rate | 9 RCTs (n = 624) | OR 1.78, 95% CI 1.26 to 2.50 | Serious | Not serious | Serious | Not serious | Not serious | Low | Risk of bias; Indirectness |
| Wang J (2021) | Acupuncture + swallowing training vs conventional rehabilitation / swallowing training | Total effective rate | 12 RCTs (n = 845) | OR 4.39, 95% CI 2.88 to 6.69 | Serious | Not serious | Not serious | Not serious | Serious | Low | Risk of bias; Publication bias |
| Xia YG (2021) | Tongue three-needle alone or tongue three-needle + co-intervention vs non-tongue three-needle control | Total effective rate | 21 RCTs (n = 1,442) | RR 1.24, 95% CI 1.18 to 1.31 | Serious | Not serious | Serious | Not serious | Not serious | Low | Risk of bias; Indirectness |
| Zhao N (2021) | Xingnao Kaiqiao acupuncture vs conventional acupuncture | Total effective rate | 4 RCTs (n = 346) | RR 1.18, 95% CI 1.09 to 1.28 | Serious | Not serious | Serious | Not serious | Not serious | Low | Risk of bias; Indirectness |
| Zhao N (2021) | Xingnao Kaiqiao acupuncture vs swallowing therapeutic apparatus | Total effective rate | 1 RCT (n = 77) | RR 1.01, 95% CI 0.80 to 1.29 | Serious | Not serious | Serious | Serious | Not serious | Very low | Risk of bias; Indirectness; Imprecision |
| Zhao N (2021) | Xingnao Kaiqiao acupuncture + rehabilitation training vs rehabilitation training | Total effective rate | 8 RCTs (n = 797) | RR 1.22, 95% CI 1.15 to 1.30 | Serious | Not serious | Serious | Not serious | Not serious | Low | Risk of bias; Indirectness |
| Zhao N (2021) | Xingnao Kaiqiao acupuncture + swallowing therapeutic apparatus/VitalStim vs swallowing therapeutic apparatus/VitalStim | Total effective rate | 4 RCTs (n = 363) | RR 1.22, 95% CI 1.11 to 1.35 | Serious | Not serious | Serious | Not serious | Not serious | Low | Risk of bias; Indirectness |
| Zhao N (2021) | Xingnao Kaiqiao acupuncture + western medicine vs western medicine | Total effective rate | 3 RCTs (n = 324) | RR 1.43, 95% CI 1.27 to 1.61 | Serious | Not serious | Serious | Not serious | Not serious | Low | Risk of bias; Indirectness |
| Tang Y (2022) | Nape acupuncture + rehabilitation training vs rehabilitation training alone | Clinical effectiveness | 9 RCTs (n = 837) | OR 3.94, 95% CI 2.57 to 6.04 | Serious | Not serious | Not serious | Not serious | Not serious | Moderate | Risk of bias |
| Wang P (2022) | Acupuncture with or without rehabilitation vs rehabilitation without acupuncture | Significant effective rate | 7 RCTs (n = 637) | RR 1.51, 95% CI 1.30 to 1.75 | Serious | Not serious | Serious | Not serious | Not serious | Low | Risk of bias; Indirectness |
| Li L (2023) | Tongue acupuncture alone or tongue acupuncture combined with other therapies vs non-tongue-acupuncture control | Total effective rate | 13 RCTs (n = 1,094) | OR 3.83, 95% CI 2.61 to 5.62 | Serious | Not serious | Serious | Not serious | Not serious | Low | Risk of bias; Indirectness |
| Li X (2023) | Electroacupuncture alone or combined with co-interventions vs matched control without EA | Clinical response rate | 12 RCTs (n = 1,151) | OR 2.63, 95% CI 1.97 to 3.53 | Serious | Not serious | Serious | Not serious | Not serious | Low | Risk of bias; Indirectness |
| Luo J (2023) | Acupuncture + balloon dilation vs balloon dilation alone | Clinical effective rate | 5 RCTs (n = 273) | OR 5.50, 95% CI 2.32 to 13.05 | Very serious | Not serious | Not serious | Not serious | Not serious | Low | Risk of bias |
| Si HJ (2023) | Eye acupuncture alone or combined with co-intervention vs non-eye-acupuncture control | Cure rate | 8 RCTs (n = 658) | OR 2.14, 95% CI 1.52 to 2.99 | Serious | Not serious | Serious | Not serious | Serious | Low | Risk of bias; Indirectness; Publication bias |
| Si HJ (2023) | Eye acupuncture alone or combined with co-intervention vs non-eye-acupuncture control | Total effective rate | 13 RCTs (n = 1,084) | OR 4.34, 95% CI 2.92 to 6.44 | Serious | Not serious | Serious | Not serious | Serious | Low | Risk of bias; Indirectness; Publication bias |
| Wang JP (2023) | Intradermal needle + co-intervention vs the same co-intervention alone | Total effective rate | 9 RCTs (n = 690) | OR 4.29, 95% CI 2.70 to 6.82 | Serious | Not serious | Serious | Not serious | Serious | Low | Risk of bias; Indirectness; Publication bias |
| Zuo TX (2023) | Acupuncture + rTMS + usual rehabilitation vs usual rehabilitation | Clinical total effective rate | 8 RCTs (n = 452) | OR 4.71, 95% CI 2.68 to 8.28 | Serious | Not serious | Serious | Not serious | Not serious | Low | Risk of bias; Indirectness |
| Sun HQ (2024) | Xingnao Kaiqiao acupuncture alone or combined with co-intervention vs non-Xingnao Kaiqiao control | Total effective rate | 20 RCTs (n = 2,076) | OR 4.45, 95% CI 3.40 to 5.83 | Very serious | Not serious | Not serious | Not serious | Not serious | Low | Risk of bias |
| Wang L (2024) | TCM + acupuncture vs acupuncture and/or conventional treatment | Total effective rate | 21 RCTs (n = 1,760) | RR 1.23, 95% CI 1.18 to 1.28 | Serious | Not serious | Serious | Not serious | Not serious | Low | Risk of bias; Indirectness |
| Xu H (2024) | Acupuncture for dredging governor meridian and regulating the mind, alone or with co-intervention, vs non-study acupuncture control | Total effective rate | 8 RCTs (n = 716) | RR 1.26, 95% CI 1.17 to 1.35 | Serious | Not serious | Serious | Not serious | Not serious | Low | Risk of bias; Indirectness |

Table S7B. GRADE certainty of evidence for swallowing function (scales) outcomes

| **Study** | **Intervention/comparison** | **Specific outcome** | **No. of RCTs**  **(participants)** | **Effect estimate**  **(95% CI)** | **RoB** | **Inconsistency** | **Indirectness** | **Imprecision** | **Publication bias** | **Quality of evidence** | **Main reasons for downgrading** |
| --- | --- | --- | --- | --- | --- | --- | --- | --- | --- | --- | --- |
| Wong ISY (2012) | Acupuncture + conventional rehabilitation vs conventional rehabilitation alone | Overall swallowing outcomes | 9 RCTs (sample size not pooled) | Not pooled (narrative synthesis only) | Serious | Not serious | Serious | Not serious | Not serious | Low | Risk of bias; Indirectness |
| Zhu Y (2012) | Acupuncture or acupuncture + rehabilitation vs rehabilitation | Water swallow test rating | 2 RCTs (n = 184) | WMD -0.96, 95% CI -1.16 to -0.77 | Very serious | Not serious | Serious | Serious | Serious | Very low | Risk of bias; Indirectness; Imprecision; Publication bias |
| Yu C (2016) | Acupuncture + swallowing rehabilitation vs swallowing rehabilitation without acupuncture | Kubota Water Swallowing Test score | 3 RCTs (n = 352) | MD -0.67, 95% CI -0.79 to -0.55 | Very serious | Very serious | Not serious | Not serious | Not serious | Very low | Risk of bias; Inconsistency |
| Ye QP (2017) | Acupuncture alone or acupuncture combined with co-interventions vs rehabilitation / medication / sham or other non-acupuncture controls | Caiteng 7 Rank (CT7R) | 2 RCTs (n not clearly reported) | RR 1.22, 95% CI 1.04 to 1.42 | Very serious | Not serious | Serious | Serious | Not serious | Very low | Risk of bias; Indirectness; Imprecision |
| Ye QP (2017) | Acupuncture alone or acupuncture combined with co-interventions vs rehabilitation / medication / sham or other non-acupuncture controls | Standard Swallowing Assessment (SSA) | 11 RCTs (n not clearly reported) | MD -3.70, 95% CI -4.93 to -2.48 | Very serious | Serious | Serious | Not serious | Not serious | Very low | Risk of bias; Inconsistency; Indirectness |
| Ye QP (2017) | Acupuncture alone or acupuncture combined with co-interventions vs rehabilitation / medication / sham or other non-acupuncture controls | Swallowing function score | 8 RCTs (n not clearly reported) | MD 1.48, 95% CI 1.18 to 1.79 | Very serious | Serious | Serious | Not serious | Not serious | Very low | Risk of bias; Inconsistency; Indirectness |
| Ye QP (2017) | Acupuncture alone or acupuncture combined with co-interventions vs rehabilitation / medication / sham or other non-acupuncture controls | Watian Swallowing Test (WST) score | 15 RCTs (n not clearly reported) | Reported as lower WST score; 95% CI -1.11 to -0.47 | Very serious | Serious | Serious | Not serious | Not serious | Very low | Risk of bias; Inconsistency; Indirectness |
| Zhang SH (2017) | Electroacupuncture or electroacupuncture + swallowing training vs control | Water swallow test | 4 RCTs (n = 276) | MD -1.04, 95% CI -1.28 to -0.80 | Very serious | Serious | Serious | Not serious | Serious | Very low | Risk of bias; Inconsistency; Indirectness; Publication bias |
| Zhang Y (2017) | Electroacupuncture or electroacupuncture + co-intervention vs control | Kubota water swallow test | 3 RCTs (n = 200) | WMD 1.07, 95% CI 0.46 to 1.69 | Very serious | Very serious | Serious | Not serious | Serious | Very low | Risk of bias; Inconsistency; Indirectness; Publication bias |
| Li LX (2019) | Acupuncture + swallowing training vs swallowing training alone | Swallowing function assessment (SFA) | 5 RCTs (n not clearly reported) | SMD 1.40, 95% CI 1.18 to 1.61 | Serious | Serious | Not serious | Not serious | Not serious | Low | Risk of bias; Inconsistency |
| Tang Q (2019) | Acupuncture + rehabilitation training vs rehabilitation training / sham acupuncture + rehabilitation | SSA score | 6 RCTs (n = 537) | MD -3.20, 95% CI -3.78 to -2.61 | Serious | Not serious | Not serious | Not serious | Serious | Low | Risk of bias; Publication bias |
| Tang Q (2019) | Acupuncture + rehabilitation training vs rehabilitation training / sham acupuncture + rehabilitation | Water swallow test score | 7 RCTs (n = 658) | MD -0.65, 95% CI -0.91 to -0.39 | Serious | Very serious | Not serious | Not serious | Serious | Very low | Risk of bias; Inconsistency; Publication bias |
| Tang XR (2019) | Acupuncture + rehabilitation therapy vs rehabilitation alone and/or conventional western medicine | Dysphagia clinical scale score | 7 RCTs (n = 605) | MD 2.01, 95% CI 1.78 to 2.24 | Serious | Not serious | Serious | Not serious | Not serious | Low | Risk of bias; Indirectness |
| Tang XR (2019) | Acupuncture + rehabilitation therapy vs rehabilitation alone and/or conventional western medicine | Kubota Water Swallowing Test score | 4 RCTs (n = 360) | MD -0.66, 95% CI -0.78 to -0.53 | Serious | Not serious | Serious | Not serious | Not serious | Low | Risk of bias; Indirectness |
| Wang H (2019) | Acupuncture + rehabilitation training vs swallowing training | Fujishima ingestion-swallowing function grade | 5 RCTs (n = 361) | MD 2.00, 95% CI 1.80 to 2.20 | Serious | Not serious | Serious | Not serious | Not serious | Low | Risk of bias; Indirectness |
| Wang H (2019) | Acupuncture + rehabilitation training vs swallowing training | SSA score | 2 RCTs (n = 161) | MD -5.98, 95% CI -7.04 to -4.91 | Serious | Not serious | Not serious | Serious | Not serious | Low | Risk of bias; Imprecision |
| Wang H (2019) | Acupuncture + rehabilitation training vs swallowing training | Water swallow test score | 4 RCTs (n = 367) | MD -0.80, 95% CI -0.88 to -0.72 | Serious | Not serious | Serious | Not serious | Not serious | Low | Risk of bias; Indirectness |
| Xu LJ (2019) | Xingnao Kaiqiao acupuncture alone or combined with co-intervention vs non-Xingnao Kaiqiao control | FIRS | 3 RCTs (n = 204) | 0.44 higher, 95% CI 0.18 higher to 0.70 higher | Very serious | Not serious | Not serious | Not serious | Not serious | Low | Risk of bias |
| Xu LJ (2019) | Xingnao Kaiqiao acupuncture alone or combined with co-intervention vs non-Xingnao Kaiqiao control | SSA | 4 RCTs (n = 394) | 3.49 lower, 95% CI 4.20 lower to 2.78 lower | Very serious | Not serious | Not serious | Not serious | Not serious | Low | Risk of bias |
| Xu LJ (2019) | Xingnao Kaiqiao acupuncture alone or combined with co-intervention vs non-Xingnao Kaiqiao control | WST | 9 RCTs (n = 832) | 0.67 lower, 95% CI 0.74 lower to 0.60 lower | Very serious | Not serious | Not serious | Not serious | Not serious | Low | Risk of bias |
| Huang J (2020) | Electroacupuncture + swallowing rehabilitation training vs swallowing rehabilitation training alone | Ichiro Fujishima Rating Scale (IFRS) | 2 RCTs (n = 228) | MD 1.94, 95% CI 1.67 to 2.22 | Serious | Very serious | Not serious | Not serious | Not serious | Very low | Risk of bias; Inconsistency |
| Huang J (2020) | Electroacupuncture + swallowing rehabilitation training vs swallowing rehabilitation training alone | Water Swallowing Test (WST) | 3 RCTs (n = 196) | MD -0.78, 95% CI -1.07 to -0.50 | Serious | Serious | Serious | Not serious | Not serious | Very low | Risk of bias; Inconsistency; Indirectness |
| Tan SY (2020) | Acupuncture + swallowing function training vs conventional treatment / swallowing training | Kubota Water Swallowing Test score | 4 RCTs (n = 328) | SMD -0.47, 95% CI -2.23 to 1.39 | Serious | Very serious | Not serious | Serious | Not serious | Very low | Risk of bias; Inconsistency; Imprecision |
| Geng JJ (2021) | Electroacupuncture + swallowing training vs swallowing training | Fujishima Ichiro rating scale | 2 RCTs (n = 228) | MD 2.09, 95% CI 0.65 to 3.53 | Serious | Very serious | Serious | Not serious | Not serious | Very low | Risk of bias; Inconsistency; Indirectness |
| Geng JJ (2021) | Electroacupuncture + swallowing training vs swallowing training | Kubota water swallow test | 2 RCTs (n = 126) | MD -0.54, 95% CI -0.88 to -0.20 | Serious | Not serious | Serious | Not serious | Not serious | Low | Risk of bias; Indirectness |
| Hu SY (2021) | Tongue three-needle alone or tongue three-needle + co-intervention vs non-tongue three-needle control | Water swallow test score | 2 RCTs (n = 165) | MD -0.85, 95% CI -1.40 to -0.30 | Serious | Serious | Serious | Not serious | Not serious | Very low | Risk of bias; Inconsistency; Indirectness |
| Li (2021) | Acupuncture vs control | Kubota water swallow grade | 11 RCTs (n = 912) | WMD -0.69, 95% CI -0.78 to -0.60 | Serious | Not serious | Not serious | Not serious | Serious | Low | Risk of bias; Publication bias |
| Li (2021) | Acupuncture vs control | SSA | 6 RCTs (n = 586) | WMD -3.41, 95% CI -3.98 to -2.84 | Serious | Not serious | Not serious | Not serious | Serious | Low | Risk of bias; Publication bias |
| Lu Y (2021) | Acupuncture + swallowing rehabilitation training vs swallowing rehabilitation training | Fujishima feeding-swallowing function grade score | 3 RCTs (n = 275) | SMD 1.92, 95% CI 1.30 to 2.54 | Serious | Serious | Serious | Not serious | Not serious | Very low | Risk of bias; Inconsistency; Indirectness |
| Lu Y (2021) | Acupuncture + swallowing rehabilitation training vs swallowing rehabilitation training | SSA score | 8 RCTs (n = 680) | MD -4.63, 95% CI -5.68 to -3.59 | Serious | Serious | Serious | Not serious | Not serious | Very low | Risk of bias; Inconsistency; Indirectness |
| Lu Y (2021) | Acupuncture + swallowing rehabilitation training vs swallowing rehabilitation training | Water swallowing test score | 8 RCTs (n = 929) | MD -0.75, 95% CI -1.11 to -0.41 | Serious | Serious | Serious | Not serious | Not serious | Very low | Risk of bias; Inconsistency; Indirectness |
| Wang J (2021) | Acupuncture + swallowing training vs conventional rehabilitation / swallowing training | Kubota Water Swallowing Test score | 3 RCTs (n = 162) | MD -0.82, 95% CI -1.25 to -0.39 | Serious | Not serious | Serious | Not serious | Not serious | Low | Risk of bias; Indirectness |
| Xia YG (2021) | Tongue three-needle alone or tongue three-needle + co-intervention vs non-tongue three-needle control | SSA score | 5 RCTs (n = 292) | SMD -2.18, 95% CI -3.04 to -1.32 | Serious | Not serious | Serious | Not serious | Not serious | Low | Risk of bias; Indirectness |
| Xia YG (2021) | Tongue three-needle alone or tongue three-needle + co-intervention vs non-tongue three-needle control | Wada drinking water test score | 6 RCTs (n = 364) | SMD -1.17, 95% CI -1.29 to -1.04 | Serious | Not serious | Serious | Not serious | Not serious | Low | Risk of bias; Indirectness |
| Zhao N (2021) | Xingnao Kaiqiao acupuncture + rehabilitation training vs rehabilitation training | SSA score | 3 RCTs (n = 289) | MD 3.59, 95% CI 2.62 to 4.57 | Serious | Not serious | Not serious | Not serious | Serious | Low | Risk of bias; Publication bias |
| Zhong LD (2021) | Acupuncture alone or acupuncture combined with matched co-interventions vs non-acupuncture control with the same base treatment | Ichiro Fujishima rating scale (IFRS) score | 12 RCTs (n not clearly reported) | MD 1.68, 95% CI 1.16 to 2.20 | Serious | Serious | Serious | Not serious | Not serious | Very low | Risk of bias; Inconsistency; Indirectness |
| Zhong LD (2021) | Acupuncture alone or acupuncture combined with matched co-interventions vs non-acupuncture control with the same base treatment | Standardized swallowing assessment (SSA) score | 13 RCTs (n = 1,204) | MD -3.78, 95% CI -4.64 to -2.91 | Serious | Serious | Serious | Not serious | Not serious | Very low | Risk of bias; Inconsistency; Indirectness |
| Zhong LD (2021) | Acupuncture alone or acupuncture combined with matched co-interventions vs non-acupuncture control with the same base treatment | Watian swallowing test (WST) score | 11 RCTs (n not clearly reported) | MD -1.21, 95% CI -1.85 to -0.57 | Serious | Serious | Serious | Not serious | Not serious | Very low | Risk of bias; Inconsistency; Indirectness |
| Jiang (2022) | Manual acupuncture vs rehabilitation | WST | 5 RCTs (n = 292) | MD 0.47 lower, 95% CI 0.72 lower to 0.23 lower | Serious | Not serious | Not serious | Not serious | Serious | Low | Risk of bias; Publication bias |
| Jiang (2022) | Manual acupuncture + rehabilitation vs rehabilitation | WST | 16 RCTs (n = 1,220) | MD 0.74 lower, 95% CI 0.96 lower to 0.52 lower | Serious | Not serious | Not serious | Not serious | Not serious | Moderate | Risk of bias |
| Tang Y (2022) | Nape acupuncture + rehabilitation training vs rehabilitation training alone | SSA score | 9 RCTs (n = 797) | WMD -2.57, 95% CI -3.51 to -1.62 | Serious | Serious | Not serious | Not serious | Serious | Very low | Risk of bias; Inconsistency; Publication bias |
| Li L (2023) | Tongue acupuncture alone or tongue acupuncture combined with other therapies vs non-tongue-acupuncture control | SSA score | 7 RCTs (n = 755) | MD -4.74, 95% CI -5.44 to -4.04 | Serious | Serious | Serious | Not serious | Not serious | Very low | Risk of bias; Inconsistency; Indirectness |
| Li L (2023) | Tongue acupuncture alone or tongue acupuncture combined with other therapies vs non-tongue-acupuncture control | WST score | 6 RCTs (n = 389) | MD -0.56, 95% CI -1.23 to 0.12 | Serious | Serious | Serious | Serious | Not serious | Very low | Risk of bias; Inconsistency; Indirectness; Imprecision |
| Li X (2023) | Electroacupuncture alone or combined with co-interventions vs matched control without EA | SSA score (treatment end) | 6 RCTs (n = 595) | MD -3.11, 95% CI -6.45 to 0.23 | Serious | Not serious | Serious | Serious | Not serious | Very low | Risk of bias; Indirectness; Imprecision |
| Luo J (2023) | Acupuncture + balloon dilation vs balloon dilation alone | SSA score | 4 RCTs (n = 216) | MD -4.22, 95% CI -4.57 to -3.87 | Very serious | Not serious | Not serious | Not serious | Not serious | Low | Risk of bias |
| Si HJ (2023) | Eye acupuncture alone or combined with co-intervention vs non-eye-acupuncture control | Standard swallowing function scale score | 2 RCTs (n = 164) | MD -10.70, 95% CI -15.56 to -5.84 | Serious | Very serious | Not serious | Serious | Serious | Very low | Risk of bias; Inconsistency; Imprecision; Publication bias |
| Si HJ (2023) | Eye acupuncture alone or combined with co-intervention vs non-eye-acupuncture control | WST score | 9 RCTs (n = 739) | MD -0.85, 95% CI -1.26 to -0.43 | Serious | Very serious | Not serious | Not serious | Serious | Very low | Risk of bias; Inconsistency; Publication bias |
| Wang JP (2023) | Intradermal needle + co-intervention vs the same co-intervention alone | SSA score | 4 RCTs (n = 368) | MD -3.46, 95% CI -4.61 to -2.30 | Serious | Serious | Serious | Not serious | Serious | Very low | Risk of bias; Inconsistency; Indirectness; Publication bias |
| Wang JP (2023) | Intradermal needle + co-intervention vs the same co-intervention alone | WST grade | 6 RCTs (n = 470) | MD -0.63, 95% CI -0.84 to -0.42 | Serious | Serious | Serious | Not serious | Serious | Very low | Risk of bias; Inconsistency; Indirectness; Publication bias |
| Zuo TX (2023) | Acupuncture + rTMS + usual rehabilitation vs usual rehabilitation | FOIS score | 3 RCTs (n = 197) | MD 0.92, 95% CI 0.59 to 1.26 | Serious | Not serious | Not serious | Serious | Not serious | Low | Risk of bias; Imprecision |
| Zuo TX (2023) | Acupuncture + rTMS + usual rehabilitation vs usual rehabilitation | SSA score | 6 RCTs (n = 318) | MD -6.57, 95% CI -9.71 to -3.42 | Serious | Very serious | Not serious | Not serious | Not serious | Very low | Risk of bias; Inconsistency |
| Zuo TX (2023) | Acupuncture + rTMS + usual rehabilitation vs usual rehabilitation | WST score | 9 RCTs (n = 461) | MD -1.17, 95% CI -1.57 to -0.76 | Serious | Very serious | Not serious | Not serious | Not serious | Very low | Risk of bias; Inconsistency |
| Sun HQ (2024) | Xingnao Kaiqiao acupuncture alone or combined with co-intervention vs non-Xingnao Kaiqiao control | SSA | 9 RCTs (n = 758) | SMD -1.14, 95% CI -1.42 to -0.87 | Very serious | Serious | Not serious | Not serious | Serious | Very low | Risk of bias; Inconsistency; Publication bias |
| Sun HQ (2024) | Xingnao Kaiqiao acupuncture alone or combined with co-intervention vs non-Xingnao Kaiqiao control | WST | 11 RCTs (n = 1,360) | SMD -2.73, 95% CI -3.53 to -1.92 | Very serious | Very serious | Not serious | Not serious | Serious | Very low | Risk of bias; Inconsistency; Publication bias |
| Wang L (2024) | TCM + acupuncture vs acupuncture and/or conventional treatment | Kubota Water Swallowing Test score | 10 RCTs (n = 822) | MD -0.79, 95% CI -0.96 to -0.62 | Serious | Serious | Serious | Not serious | Not serious | Very low | Risk of bias; Inconsistency; Indirectness |
| Wang L (2024) | TCM + acupuncture vs acupuncture and/or conventional treatment | SSA score | 5 RCTs (n = 427) | MD -4.84, 95% CI -6.39 to -3.29 | Serious | Serious | Not serious | Not serious | Not serious | Low | Risk of bias; Inconsistency |
| Xu H (2024) | Acupuncture for dredging governor meridian and regulating the mind, alone or with co-intervention, vs non-study acupuncture control | SSA score | 7 RCTs (n = 596) | MD -3.89, 95% CI -4.68 to -3.11 | Serious | Serious | Not serious | Not serious | Not serious | Low | Risk of bias; Inconsistency |
| Xu H (2024) | Acupuncture for dredging governor meridian and regulating the mind, alone or with co-intervention, vs non-study acupuncture control | Water swallowing test score | 3 RCTs (n = 294) | MD -1.61, 95% CI -2.20 to -1.02 | Serious | Serious | Serious | Not serious | Not serious | Very low | Risk of bias; Inconsistency; Indirectness |
| Zhang JL (2025) | Acupuncture + rehabilitation therapy vs rehabilitation therapy alone | SSA score | 12 RCTs (n = 1,096) | MD -3.64, 95% CI -4.72 to -2.56 | Serious | Serious | Not serious | Not serious | Not serious | Low | Risk of bias; Inconsistency |
| Zhang JL (2025) | Acupuncture + rehabilitation therapy vs rehabilitation therapy alone | WST score | 10 RCTs (n = 774) | MD -0.72, 95% CI -0.96 to -0.47 | Serious | Serious | Not serious | Not serious | Not serious | Low | Risk of bias; Inconsistency |

Table S7C. GRADE certainty of evidence for instrumental assessment outcomes

| **Study** | **Intervention/comparison** | **Specific outcome** | **No. of RCTs**  **(participants)** | **Effect estimate**  **(95% CI)** | **RoB** | **Inconsistency** | **Indirectness** | **Imprecision** | **Publication bias** | **Quality of evidence** | **Main reasons for downgrading** |
| --- | --- | --- | --- | --- | --- | --- | --- | --- | --- | --- | --- |
| Zhang SH (2017) | Electroacupuncture or electroacupuncture + swallowing training vs control | VFSS | 2 RCTs (n = 120) | MD 1.02, 95% CI 0.37 to 1.68 | Serious | Not serious | Not serious | Serious | Serious | Very low | Risk of bias; Imprecision; Publication bias |
| Tang Q (2019) | Acupuncture + rehabilitation training vs rehabilitation training / sham acupuncture + rehabilitation | VFSS score | 8 RCTs (n = 654) | MD 2.31, 95% CI 1.75 to 2.87 | Serious | Serious | Not serious | Not serious | Serious | Very low | Risk of bias; Inconsistency; Publication bias |
| Huang J (2020) | Electroacupuncture + swallowing rehabilitation training vs swallowing rehabilitation training alone | Video fluoroscopic swallowing study (VFSS) | 2 RCTs (n = 150) | MD 1.47, 95% CI 1.11 to 1.84 | Serious | Not serious | Not serious | Serious | Not serious | Low | Risk of bias; Imprecision |
| Tan SY (2020) | Acupuncture + swallowing function training vs conventional treatment / swallowing training | VFSS score | 4 RCTs (n = 315) | MD 1.54, 95% CI 0.96 to 2.13 | Serious | Serious | Not serious | Not serious | Serious | Very low | Risk of bias; Inconsistency; Publication bias |
| Geng JJ (2021) | Electroacupuncture + swallowing training vs swallowing training | VFSS | 2 RCTs (n = 150) | MD 1.92, 95% CI 1.51 to 2.33 | Serious | Not serious | Not serious | Serious | Not serious | Low | Risk of bias; Imprecision |
| Hu SY (2021) | Tongue three-needle alone or tongue three-needle + co-intervention vs non-tongue three-needle control | VFSS score | 2 RCTs (n = 158) | MD 2.68, 95% CI 0.51 to 4.85 | Serious | Serious | Not serious | Serious | Not serious | Very low | Risk of bias; Inconsistency; Imprecision |
| Lu Y (2021) | Acupuncture + swallowing rehabilitation training vs swallowing rehabilitation training | VFSS score | 5 RCTs (n = 354) | MD 2.53, 95% CI 1.89 to 3.17 | Serious | Serious | Not serious | Not serious | Not serious | Low | Risk of bias; Inconsistency |
| Zhong LD (2021) | Acupuncture alone or acupuncture combined with matched co-interventions vs non-acupuncture control with the same base treatment | Videofluoroscopic swallowing study (VFSS) score | 8 RCTs (n not clearly reported) | MD 2.26, 95% CI 1.77 to 2.74 | Serious | Serious | Not serious | Not serious | Not serious | Low | Risk of bias; Inconsistency |
| Jiang (2022) | Manual acupuncture + rehabilitation vs rehabilitation | VFSS | 9 RCTs (n = 690) | MD 1.35 higher, 95% CI 1.00 higher to 1.71 higher | Serious | Not serious | Not serious | Not serious | Not serious | Moderate | Risk of bias |
| Tang Y (2022) | Nape acupuncture + rehabilitation training vs rehabilitation training alone | VFSS score | 4 RCTs (n = 332) | WMD 1.33, 95% CI 1.09 to 1.58 | Serious | Serious | Serious | Serious | Serious | Very low | Risk of bias; Inconsistency; Indirectness; Imprecision; Publication bias |
| Li X (2023) | Electroacupuncture alone or combined with co-interventions vs matched control without EA | PAS score | 3 RCTs (n = 204) | MD -0.68, 95% CI -2.78 to 1.41 | Serious | Not serious | Not serious | Serious | Not serious | Low | Risk of bias; Imprecision |
| Li X (2023) | Electroacupuncture alone or combined with co-interventions vs matched control without EA | VFSS score | 3 RCTs (n = 297) | MD 0.73, 95% CI 0.29 to 1.16 | Serious | Not serious | Not serious | Serious | Not serious | Low | Risk of bias; Imprecision |
| Luo J (2023) | Acupuncture + balloon dilation vs balloon dilation alone | VFSS score | 7 RCTs (n = 350) | MD 1.53, 95% CI 1.32 to 1.75 | Very serious | Not serious | Not serious | Not serious | Not serious | Low | Risk of bias |
| Si HJ (2023) | Eye acupuncture alone or combined with co-intervention vs non-eye-acupuncture control | VFSS score | 2 RCTs (n = 184) | MD 2.33, 95% CI 0.83 to 3.83 | Serious | Very serious | Not serious | Serious | Serious | Very low | Risk of bias; Inconsistency; Imprecision; Publication bias |
| Zuo TX (2023) | Acupuncture + rTMS + usual rehabilitation vs usual rehabilitation | PAS score | 3 RCTs (n = 184) | MD -1.05, 95% CI -1.31 to -0.80 | Serious | Not serious | Not serious | Serious | Not serious | Low | Risk of bias; Imprecision |
| Zuo TX (2023) | Acupuncture + rTMS + usual rehabilitation vs usual rehabilitation | VFSS score | 4 RCTs (n = 253) | MD 1.42, 95% CI 0.83 to 2.01 | Serious | Serious | Not serious | Serious | Not serious | Very low | Risk of bias; Inconsistency; Imprecision |
| Sun HQ (2024) | Xingnao Kaiqiao acupuncture alone or combined with co-intervention vs non-Xingnao Kaiqiao control | VFSS score | 4 RCTs (n = 506) | SMD 4.89, 95% CI 2.80 to 6.97 | Very serious | Very serious | Not serious | Serious | Serious | Very low | Risk of bias; Inconsistency; Imprecision; Publication bias |
| Xu H (2024) | Acupuncture for dredging governor meridian and regulating the mind, alone or with co-intervention, vs non-study acupuncture control | VFSS score | 4 RCTs (n = 373) | MD 1.38, 95% CI 1.09 to 1.66 | Serious | Not serious | Not serious | Not serious | Serious | Low | Risk of bias; Publication bias |
| Zhang JL (2025) | Acupuncture + rehabilitation therapy vs rehabilitation therapy alone | VFSS score | 9 RCTs (n = 697) | MD 1.49, 95% CI 0.89 to 2.09 | Serious | Serious | Not serious | Not serious | Not serious | Low | Risk of bias; Inconsistency |

Table S7D. GRADE certainty of evidence for quality of life and functional recovery outcomes

| **Study** | **Intervention/comparison** | **Specific outcome** | **No. of RCTs**  **(participants)** | **Effect estimate (95% CI)** | **RoB** | **Inconsistency** | **Indirectness** | **Imprecision** | **Publication bias** | **Quality of evidence** | **Main reasons for downgrading** |
| --- | --- | --- | --- | --- | --- | --- | --- | --- | --- | --- | --- |
| Ye QP (2017) | Acupuncture alone or acupuncture combined with co-interventions vs rehabilitation / medication / sham or other non-acupuncture controls | Activities of Daily Living (ADL/Barthel/MBI) | 5 RCTs (n = 435) | MD 7.46, 95% CI 5.49 to 9.47 | Very serious | Not serious | Serious | Not serious | Not serious | Very low | Risk of bias; Indirectness |
| Ye QP (2017) | Acupuncture alone or acupuncture combined with co-interventions vs rehabilitation / medication / sham or other non-acupuncture controls | SWAL-QOL | 5 RCTs (n = 340) | MD -0.89 (-1.13 to -0.66) after sensitivity analysis; original pooled analysis non-informative with I²=100% | Very serious | Very serious | Not serious | Serious | Not serious | Very low | Risk of bias; Inconsistency; Imprecision |
| Li LX (2019) | Acupuncture + swallowing training vs swallowing training alone | Modified Barthel Index (MBI) | 1 RCT (n = 124) | SMD 1.47, 95% CI 1.07 to 1.87 | Serious | Not serious | Not serious | Serious | Not serious | Low | Risk of bias; Imprecision |
| Li LX (2019) | Acupuncture + swallowing training vs swallowing training alone | Swallowing-related quality of life (S-QOL) | 2 RCTs (n = 237) | SMD 1.06, 95% CI -0.04 to 2.17 | Serious | Not serious | Not serious | Serious | Not serious | Low | Risk of bias; Imprecision |
| Wang H (2019) | Acupuncture + rehabilitation training vs swallowing training | SWAL-QOL score | 2 RCTs (n = 161) | MD 14.50, 95% CI 8.64 to 20.36 | Serious | Not serious | Not serious | Serious | Not serious | Low | Risk of bias; Imprecision |
| Zhao DF (2019) | Acupuncture-based treatment (alone or combined with rehabilitation/drugs) vs non-acupuncture regular care | MBI score | 2 RCTs (n = 207) | WMD 12.36, 95% CI 9.17 to 15.55 | Serious | Not serious | Not serious | Serious | Not serious | Low | Risk of bias; Imprecision |
| Zhao DF (2019) | Acupuncture-based treatment (alone or combined with rehabilitation/drugs) vs non-acupuncture regular care | SWAL-QOL score | 4 RCTs (n = 381) | WMD 15.31, 95% CI 11.98 to 18.64 | Serious | Not serious | Not serious | Serious | Not serious | Low | Risk of bias; Imprecision |
| Lu Y (2021) | Acupuncture + swallowing rehabilitation training vs swallowing rehabilitation training | SWAL-QOL score | 5 RCTs (n = 477) | SMD 2.02, 95% CI 0.82 to 3.22 | Serious | Serious | Not serious | Not serious | Not serious | Low | Risk of bias; Inconsistency |
| Zhao N (2021) | Xingnao Kaiqiao acupuncture + rehabilitation training vs rehabilitation training | SWAL-QOL score | 2 RCTs (n = 221) | MD 22.30, 95% CI 0.64 to 43.96 | Serious | Very serious | Not serious | Serious | Serious | Very low | Risk of bias; Inconsistency; Imprecision; Publication bias |
| Li X (2023) | Electroacupuncture alone or combined with co-interventions vs matched control without EA | SWAL-QOL score (treatment end) | 4 RCTs (n = 252) | MD 13.24, 95% CI -7.74 to 34.21 | Serious | Not serious | Not serious | Serious | Not serious | Low | Risk of bias; Imprecision |
| Wang JP (2023) | Intradermal needle + co-intervention vs the same co-intervention alone | SWAL-QOL score | 3 RCTs (n = 179) | MD 22.07, 95% CI 2.93 to 41.20 | Serious | Serious | Not serious | Serious | Serious | Very low | Risk of bias; Inconsistency; Imprecision; Publication bias |
| Sun HQ (2024) | Xingnao Kaiqiao acupuncture alone or combined with co-intervention vs non-Xingnao Kaiqiao control | SWAL-QOL | 6 RCTs (n = 636) | SMD 1.30, 95% CI 0.87 to 1.72 | Very serious | Serious | Not serious | Not serious | Serious | Very low | Risk of bias; Inconsistency; Publication bias |
| Wang L (2024) | TCM + acupuncture vs acupuncture and/or conventional treatment | Barthel Index score | 5 RCTs (n = 370) | MD 6.80, 95% CI 3.18 to 10.41 | Serious | Serious | Not serious | Not serious | Not serious | Low | Risk of bias; Inconsistency |
| Zhang JL (2025) | Acupuncture + rehabilitation therapy vs rehabilitation therapy alone | SWAL-QOL score | 8 RCTs (n = 793) | MD 16.56, 95% CI 9.94 to 23.18 | Serious | Serious | Not serious | Not serious | Not serious | Low | Risk of bias; Inconsistency |

Table S7E. GRADE certainty of evidence for aspiration / pneumonia outcomes

| **Study** | **Intervention/comparison** | **Specific outcome** | **No. of RCTs**  **(participants)** | **Effect estimate**  **(95% CI)** | **RoB** | **Inconsistency** | **Indirectness** | **Imprecision** | **Publication bias** | **Quality of evidence** | **Main reasons for downgrading** |
| --- | --- | --- | --- | --- | --- | --- | --- | --- | --- | --- | --- |
| Chen J (2015) | Xingnao Kaiqiao acupuncture + usual care vs usual care | Pulmonary infection rate | 3 RCTs (n = 345) | RR 0.43, 95% CI 0.20 to 0.93 | Serious | Serious | Not serious | Serious | Serious | Very low | Risk of bias; Inconsistency; Imprecision; Publication bias |
| Zhao DF (2019) | Acupuncture-based treatment (alone or combined with rehabilitation/drugs) vs non-acupuncture regular care | Pulmonary infection rate | 3 RCTs (n = 343) | OR 0.64, 95% CI 0.33 to 1.23 | Serious | Not serious | Not serious | Serious | Not serious | Low | Risk of bias; Imprecision |
| Huang J (2020) | Electroacupuncture + swallowing rehabilitation training vs swallowing rehabilitation training alone | Incidence of aspiration pneumonia (IAP) | 2 RCTs (n = 170) | OR 0.20, 95% CI 0.06 to 0.61 | Serious | Not serious | Not serious | Serious | Not serious | Low | Risk of bias; Imprecision |
| Li (2021) | Acupuncture vs control | Pulmonary infection rate | 2 RCTs (n = 283) | RR 0.70, 95% CI 0.43 to 1.12 | Serious | Not serious | Not serious | Not serious | Serious | Low | Risk of bias; Publication bias |

Table S7F. GRADE certainty of evidence for safety / adverse events outcomes

| **Study** | **Intervention/comparison** | **Specific outcome** | **No. of RCTs (participants)** | **Effect estimate (95% CI)** | **RoB** | **Inconsistency** | **Indirectness** | **Imprecision** | **Publication bias** | **Quality of evidence** | **Main reasons for downgrading** |
| --- | --- | --- | --- | --- | --- | --- | --- | --- | --- | --- | --- |
| Geng JJ (2021) | Electroacupuncture + swallowing training vs swallowing training | Adverse events | 2 RCTs (n = 174) | OR 2.38, 95% CI 0.15 to 37.18 | Serious | Serious | Not serious | Very serious | Not serious | Very low | Risk of bias; Inconsistency; Imprecision |
| Li X (2023) | Electroacupuncture alone or combined with co-interventions vs matched control without EA | Adverse events | 5 RCTs (n = 721) | OR 1.58, 95% CI 0.73 to 3.38 | Serious | Not serious | Not serious | Serious | Not serious | Low | Risk of bias; Imprecision |
| Zhang JL (2025) | Acupuncture + rehabilitation therapy vs rehabilitation therapy alone | Adverse events | 5 RCTs (n = 576) | RR 1.23, 95% CI 0.70 to 2.17 | Serious | Not serious | Not serious | Not serious | Not serious | Moderate | Risk of bias |

Table S7G. Operational criteria for GRADE downgrading decisions

| **GRADE domain** | **Operational criteria** |
| --- | --- |
| **Risk of bias** | Downgraded by one level when important methodological limitations in the contributing RCTs were likely to reduce confidence in the effect estimate. Downgraded by two levels when such limitations were pervasive or judged likely to substantially compromise the estimate. Particular attention was given to random sequence generation, allocation concealment, blinding, incomplete outcome data, and selective reporting. |
| **Inconsistency** | Downgraded by one level when clinically relevant unexplained heterogeneity or inconsistency in effect estimates was present. Downgraded by two levels when heterogeneity was substantial and persistent, particularly when accompanied by markedly inconsistent effect sizes or directions. Statistical heterogeneity, overlap of confidence intervals, and consistency of effect direction were considered jointly. |
| **Indirectness** | Downgraded when the population, intervention, comparator, outcome definition, or clinical context differed meaningfully from the review question. Non-standardized composite outcomes with limited direct clinical interpretability were also considered when judging indirectness. |
| **Imprecision** | Downgraded by one level when the available evidence was based on limited sample size/events or when confidence intervals indicated important uncertainty. Downgraded by two levels when data were very sparse or confidence intervals were sufficiently wide to include substantially different clinical conclusions. |
| **Publication bias** | Downgraded when outcome-specific evidence indicated possible publication bias, such as funnel-plot asymmetry, statistical evidence of small-study effects, or other clear indications of selective dissemination. Absence of a formal publication-bias test alone was not considered sufficient for downgrading. |

Table S7H. Inter-rater agreement for independent GRADE assessments before consensus

| **Assessment** | **No. of paired assessments** | **Exact agreement, %** | **Linearly weighted Cohen's κ** |
| --- | --- | --- | --- |
| **Overall GRADE certainty** | **162** | **68.5** | **0.456** |
| **Risk of bias** | **162** | **95.7** | **0.885** |
| **Inconsistency** | **162** | **77.2** | **0.690** |
| **Indirectness** | **162** | **70.4** | **0.406** |
| **Imprecision** | **162** | **88.3** | **0.695** |
| **Publication bias** | **162** | **72.2** | **0.254** |

Note. Agreement was calculated using the two reviewers' independent assessments before consensus. Disagreements were subsequently resolved through discussion based on the predefined GRADE criteria, and consensus ratings were used in the final analyses.

****Table S8.**** Detailed outcome-domain coverage

| **Outcome domain** | **Operational definition** | **Common instruments / tools** | **SRs/MAs reporting this domain (any outcome), n**  **(%)** | **SRs/MAs reporting this domain as primary outcome, n**  **(%)** |
| --- | --- | --- | --- | --- |
| Effective rate | Composite 'effective rate' outcome | NA | 45 (100.0%) | 16 (35.6%) |
| Swallowing function (scales) | Clinical swallowing function scales | SSA; WST; FOIS; other scales | 39 (86.7%) | 10 (22.2%) |
| Instrumental assessment (VFSS/FEES/PAS) | Instrument-based swallowing assessment | VFSS; FEES; PAS | 24 (53.3%) | 17 (37.8%) |
| Quality of life | Swallowing-related or health-related quality of life | SWAL-QOL; other QoL scales | 13 (28.9%) | 0 (0.0%) |
| Aspiration / pneumonia | Aspiration events and/or pneumonia incidence | Clinical diagnosis; reported complications | 5 (11.1%) | 2 (4.4%) |
| Safety / adverse events | Any harms/adverse events | Adverse events | 6 (13.3%) | 0 (0.0%) |
| Nutrition | Nutritional status outcomes | Weight; BMI; serum albumin | 0 (0.0%) | 0 (0.0%) |

Table S9. Threshold robustness analysis of the top 10 association rules under alternative parameter settings

| **Dimension** | **Primary thresholds by dimension (support: 10/15/20/15; confidence: 80%)** | **Alt-1 (support: 15/20/25/20; confidence: 80%)** | **Alt-2 (support: 10/15/20/15; confidence: 90%)** | **Alt-3 (support: 15/20/25/20; confidence: 90%)** | **Alt-4 (support: 10/15/20/15; confidence: 80%; lift > 1)** |
| --- | --- | --- | --- | --- | --- |
| Acupoints | 10 | 10 | 7 | 7 | 10 |
| Meridians | 10 | 5 | 4 | 2 | 10 |
| Stimulation sites | 10 | 9 | 2 | 2 | 8 |
| Specific acupoint attributes | 10 | 6 | 8 | 6 | 10 |

Supplementary Figures

****Figure S1.**** Evidence flow (Sankey diagram)

****Figure S2.**** Distribution of intervention type, comparator type, and co-intervention

**Figure S3. Heatmap of AMSTAR 2 item-level ratings with cumulative percentage distribution. Overall confidence ratings are based on the original AMSTAR 2 critical-domain configuration; results under the alternative configuration are provided in Supplementary Tables S6B and S6C.**

Figure S4. Frequency distributions of core acupoints, meridians, stimulation sites, and specific acupoint attributes
